# *ASYMMETRIC LEAVES1* and *REVOLUTA* are the key regulatory genes associated with pitcher development in *Nepenthes khasiana*

**DOI:** 10.1038/s41598-019-42779-6

**Published:** 2019-04-19

**Authors:** Jeremy Dkhar, Ashwani Pareek

**Affiliations:** Stress Physiology and Molecular Biology Laboratory, School of Life Sciences, Jawaharlal Nehru University, New Delhi, 110067 India

**Keywords:** Leaf development, Plant evolution

## Abstract

*Nepenthes* develops highly specialized insect-eating organs called pitchers that provide adequate insect-derived nutrients to the plants to offset low nutrient availability in their natural habitat. But so far, the molecular basis of *Nepenthes* pitcher development remains largely unknown. In an attempt to unravel the underlying mechanisms of pitcher formation, we made morphological observations of the developing *N*. *khasiana* leaf and performed RNA-seq to identify genes controlling pitcher development. Histology and scanning electron microscopy photomicrographs show that pitcher formation in *N*. *khasiana* occurs early in development and shares anatomical features with the young in-rolled leaf base lamina. Analysis of the RNA-seq data indicated that the modification of the leaf into a pitcher is associated with the altered expressions of leaf polarity genes *ASYMMETRIC LEAVES1* (*AS1*) and *REVOLUTA* (*REV*). In fact, both genes displayed exclusive or relatively higher expressions in the tip of the leaf that later developed into a pitcher. We propose that *NkAS1* may act to inhibit lamina outgrowth and promote the formation of the tendril. Increased *NkREV* expression may have been involved in the formation of the *N*. *khasiana* pitcher. This dataset will allow further research into this area and serve as the basis for understanding *Nepenthes* pitcher development.

## Introduction

Alterations in the expression of key regulatory genes involved in development often lead to morphological novelty^1^. How these changes in gene expression produced new morphologies during evolution remains a key question in biology. In plants, a remarkable amount of morphological innovations can be seen, some of which are manifested in the leaf. Leaves show a varying degree of forms, shapes and sizes, and these variations have been well-documented, at least in some plant species. Hay and Tsiantis^2^ investigated the genetic basis of the differences in leaf forms between two closely related plant species, *Arabidopsis thaliana* (Arabidopsis) and *Cardamine hirsuta*. The authors showed that in *C*. *hirsuta*, KNOX proteins are required to produce dissected leaf form whereas, in Arabidopsis, KNOX proteins are excluded to produce simple leaf form. Evidently, repression of *KNOX* expression by the ASYMMETRICLEAVES1/ROUGHSHEATH2/PHANTASTICA (ARP) proteins is conserved between the two species; but in *C*. *hirsuta*, this regulatory module is tinkered to control new developmental processes giving rise to a diverse leaf form^2^. The evolutionary tinkering of existing mechanisms into new ones is a common phenomenon and is most noticeable at the molecular level^3^. In some instances, tinkering involves expanding gene expression domains. Gleissberg *et al*.^4^ demonstrated that the peltate-shaped leaf of *Tropaeolum majus* evolved through localized expansion in the expression of *FILAMENTOUS FLOWER* (*FIL*), a YABBY gene specifying leaf abaxial identity. It was then thought that the evolution of the pitcher-shaped leaf of the carnivorous plant *Sarracenia purpurea* might involve a similar mechanism as observed in *T*. *majus*. Fukushima *et al*.^5^ examined the expressions of *FIL* and the adaxial identity promoting HD-ZIPIII gene *PHABULOSA* (*PHB*), and found that *FIL* and *PHB* expressions in *S*. *purpurea* do not point to their role in pitcher formation; rather, changes in the orientation of cell division led to the development of the *Sarracenia* pitcher.

Unlike *Sarracenia*, pitchers in *Nepenthes* are initiated at the tips of tendrils attached to a photosynthesizing-efficient leaf base lamina^6^. Two hypotheses have been put forward to explain the evolution of pitchers in *Nepenthes*. Juniper and Burras^7^ suggested that the *Nepenthes* pitcher appears to be an extension of the leaf midvein whereas Juniper *et al*.^8^ considered it a modification, through a process of epiascidiation that involves in-rolling of the adaxial leaf surface followed by marginal fusion. But so far, the molecular mechanism of pitcher development in *Nepenthes* has remained largely unknown.

The advent of high-throughput sequencing technologies has revolutionized research in plant biology. Its application in plant developmental biology has seen tremendous progress in recent years with rewarding results^9^. Although RNA-seq analyses have recently been reported in *Nepenthes*, these studies pertain to *de novo* assembly and annotation of the transcriptome data^10,11^. In the present study, we carried out morphological observations of the developing *N*. *khasiana* leaf and employed RNA-seq to identify differentially expressed genes enriched in each of the five developmental stages defined by the morphological changes observed. We then used reverse transcription-polymerase chain reaction (RT-PCR) to check for the local expressions of selected enriched genes in the pitcher part of the *N*. *khasiana* leaf. Our findings suggest that *AS1* together with *ERECTA* (*ER*) may act to inhibit leaf base lamina outgrowth at the tip of the *Nepenthes* leaf and promote the formation of the tendril. Pitcher formation in *N*. *khasiana* is linked to increased expression of *REV*. This study has shed light on the underlying mechanisms of *Nepenthes* pitcher development, and the identified genes controlling pitcher formation are expected to be confirmed in future.

## Results and Discussion

**Figure 1:**
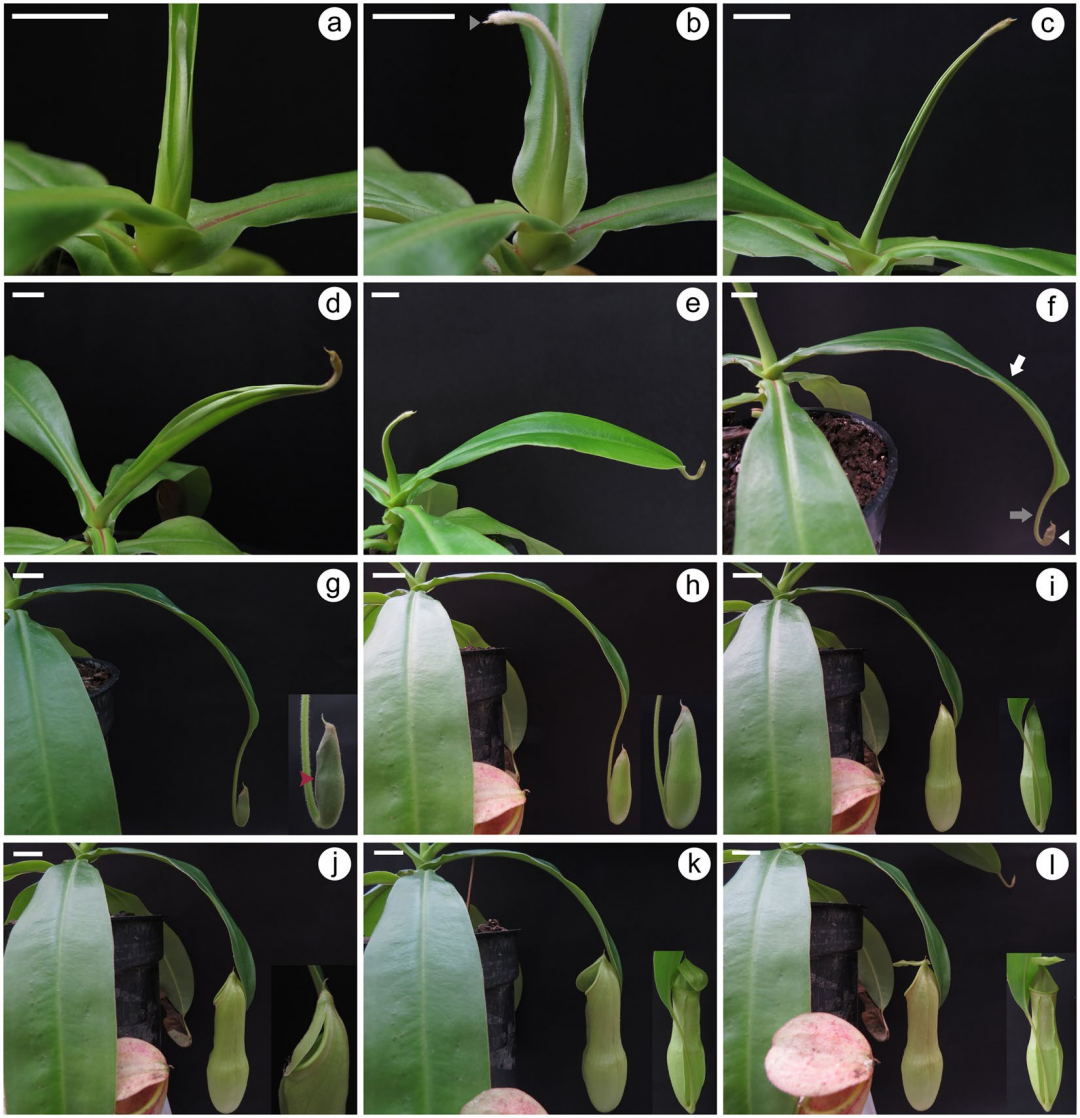
Leaf development in *N*. *khasiana*. (**a**–**c**) Leaf initiation (in **b**, grey arrowhead points to a spur). (**d**–**e**) Expansion of the leaf base and pitcher initiation. (**f**–**i**) Tendril elongation and pitcher development, expansion and elongation [in **f**, white arrow specify the leaf base, grey arrow denotes the tendril and white arrowhead depicts the pitcher (here, differentiation of the pitcher into lower digestive zone and the upper waxy zone can be seen); in **g**, protruding ‘wings’ is indicated by red arrowhead; inset shows close-up of the pitcher)]. (**j**–**l**) Pitcher maturation (inset depicts close-up of the pitcher). (**a**–**d**) bar = 1 cm; (**e**–**l**) bar = 2 cm.

Because *Nepenthes* pitchers develop at the tips of tendrils^6^, we examined growth and development of the *N*. *khasiana* leaf from the initial stages of development, visible to the naked eye (Fig. 1). Leaf development in young *N*. *khasiana* plants is initiated as a slender structure with prominent white hairs at the apex, gradually declining in density along one end of the slender structure (Fig. 1a). At this stage, the emerging leaf was seen fairly covered by the preceding leaf base lamina. After a week, the developing leaf became more apparent with increased length and the presence of a pointed structure at the apex called a ‘spur’ (Fig. 1b). The emergence of the spur in *N*. *khasiana* occurred early in development as compared to the one in *N*. *alata*^6^, but this could be due to the differences in the age of the plants studied. The developing leaf increases further in length and an opening at another end of the slender structure, lacking the visible white hairs, started appearing (Fig. 1c). At the third week, an outgrowth of the developing leaf base can be seen and the apex region begins to swell, appearing as a small, juvenile pitcher (Fig. 1d). In addition, the intervening region or ‘tendril’ separating the pitcher from the leaf base became evident at this stage. At the fifth week, leaf base lamina outgrowth continued and became flattened with a slight increase in pitcher size (Fig. 1e). Further increase in the expansion of the leaf base, tendril length and pitcher size was observed at the sixth week (Fig. 1f). Here, differentiation of the pitcher into distinct zones - digestive zone and waxy zone - was visible. As was observed by Gaume *et al*.^12^ in *N*. *alata*, a transitional zone was observed between the digestive and waxy zones of *N*. *khasiana*. At the seventh week, expansion of the leaf base ceases whereas elongation of the tendril, as well as the pitcher, continued (Fig. 1g). We also noticed the emergence of vertically-oriented structures on one side of the pitcher tube called ‘wings’ (red arrowhead in Fig. 1g), and the lid at the top of the pitcher became evident at this stage. At the eighth week, the developing leaf showed marked swelling of the pitcher tube and slight increase in tendril length (Fig. 1h). At the ninth week, a considerable increase in pitcher size was observed (Fig. 1i), but the swelling was more pronounced at the bottom of the pitcher, representing the digestive zone. The pitcher lid started detaching, initiating from the top adjacent to the spur (Fig. 1j). At this stage, the peristome can be seen appearing as a ring that surrounds the entrance of the pitcher and arranged in the form of ribs. At the tenth week, the pitcher lid was fully opened (Fig. 1k). Change in pitcher colouration started appearing, most prominently at the waxy zone moving towards the pitcher lid. At this stage, the *N*. *khasiana* leaf comprising the leaf base, tendril and the pitcher has attained maturity (Fig. 1l).

**Figure 2:**
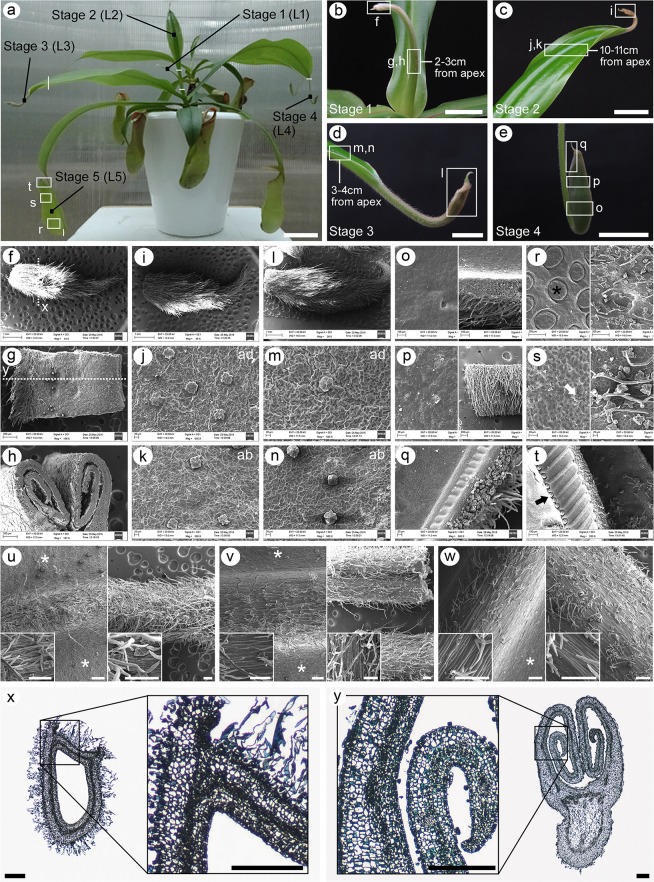
*N*. *khasiana* plant showing different stages of leaf development with their corresponding SEM and cross-sectioned images. **(a**) A shoot possessing several developing leaves, each attaining distinct stage of development: stage 1 represents the topmost leaf (L1), the leaf (L2) with the expanded leaf base is considered stage 2, the leaf (L3) showing complete expansion of the leaf base and the appearance of the pitcher characterizes stage 3, the leaf (L4) showing elongation of the tendril and expansion of the pitcher tube represents stage 4, while the leaf (L5) showing pitcher expansion with the lid remaining unopened is considered as stage 5 (white vertical/horizontal lines specify the dissected regions of each stage; bar = 6 cm). (**b**–**e**) Close-up photographs of stages 1–4 (boxes in **a**–**e** denote segments for SEM analysis; boxes are not drawn to scale; bar = 1 cm). (**f**–**t**) SEM micrographs of the different segments indicated in **a**–**e**, each segment corresponding to different regions within the different stages of *N*. *khasiana* leaf development (**j**, **m**, and left panels in **o**, **p**, **r**, **s** - adaxial (ad) surfaces; **k**, **n**, and right panels in **o**, **p**, **r**, **s** - abaxial (ab) surfaces; dashes in **f** and **g** denote regions for cross-sectioning; digestive glands are indicated by asterisk; white arrow denotes lunate cells in the waxy zone; black arrow shows the peristome nectary glands). (**u**–**w**) SEM photomicrographs of the leaf base lamina midvein (left panels) and the tendril (right panels) of the later stages of *N*. *khasiana* leaf development (Stages 3, 4 and 5). Inset shows magnified images of portions of each structure. Asterisks point to the flat leaf base abaxial lamina. bar = 200 µm; 100 µm (inset). (**x**–**y**) Cross-sectioned photomicrographs of the apex region and the in-rolled leaf base lamina. Magnified images of portions of both structures are represented in separate boxes (bar = 200 µm).

We noticed that at a particular period in the growth and development of young *N*. *khasiana* plants, developing leaves showed different developmental stages as observed in Fig. 1. In Fig. 2a, a young *N*. *khasiana* shoot can be seen developing several leaves, each attaining distinct stages of development. We considered the topmost slender leaf (L1) as stage 1 (Fig. 2a,b) while the leaf (L2) with the expanded leaf base was considered stage 2 (Fig. 2a,c). The leaf (L3) showing complete expansion of the leaf base and the appearance of the pitcher tube characterized stage 3 (Fig. 2a,d) while the leaf (L4) showing elongation of the tendril and expansion of the pitcher tube represented stage 4 (Fig. 2a,e). The leaf (L5) showing pitcher expansion with the lid remaining unopened was considered as stage 5 (Fig. 2a). We usually see this pattern continue for a considerable period of time as the plant grow, but is lost as the plant reaches a certain height. We then examined the ultrastructure of each developmental stage using scanning electron microscopy (SEM) (Fig. 2f–t). Our results show that in stage 1, the apex region is fully covered with hairs (non-glandular trichomes), as can be seen in stages 2 and 3 (Fig. 2f,i,l). The slender structure of stage 1 at 2–3 cm from the apex is covered with hairs at one end representing the midvein region, while the flat abaxial surface is covered with glandular trichomes (Fig. 2g). Glandular trichomes are also present on the adaxial and abaxial epidermal surfaces of the leaf base lamina of stages 2 and 3, respectively (Fig. 2j,k,m,n). Besides other functions, glandular and non-glandular trichomes are known to offer protection against insect herbivory (Serna and Martin^13^ and references therein). As most *Nepenthes* plants rely on insects for their nutrients, these trichomes may shield young developing leaf tissues from herbivory by visiting insects. The epidermal surfaces of the adaxial and abaxial leaf base lamina of stages 2 and 3 are made up of irregularly shaped cells (Fig. 2j,k,m,n), but in the adaxial epidermis, cells are much larger in size (Fig. 2m). As the leaf develops further to form a tiny pitcher separated from the leaf base by the tendril (stage 4), the outer epidermal surface of the pitcher is still covered with hairs while the inner epidermal surface remained smooth (Fig. 2o–q). Differentiation of the inner pitcher epidermal cells into the digestive glands, the lunate cells and the nectary glands can be seen only in stage 5 (Fig. 2r–t).

We then compared SEM images of the leaf base lamina midvein (abaxial side) and the tendril of stages 3–5, and found that both structures are characterized by long, slender epidermal cells, on top of which develop glandular and non-glandular trichomes (Fig. 2u–w). The density of both glandular and non-glandular trichomes decreases as the leaf mature. This observation suggests that the tendril is an extended structure of the midvein. Is it likely then that the *Nepenthes* pitcher represents an extension of the leaf base midvein which then expands to form the pitcher tube, as suggested by Juniper and Burras?^7^ A comparison between the epidermal cells that make up the tendril/midvein (Fig. 2u–w) and those of the outer (abaxial) epidermal layer of the pitcher (right panels of Fig. 2r,s) suggested otherwise. To confirm, we analyzed the leaf of an *in vitro* raised *Nepenthes* plantlet. The SEM images clearly define the distinction between the epidermal cells of the midvein and the pitcher (Fig. S1, Supplementary information).

Figure 2h shows that the leaf base lamina outgrowth occurred prior to stage 1, but remained rolled in. In-rolling of the adaxial leaf surface followed by marginal fusion was proposed as one of the mechanisms adopted by *Nepenthes* plants to modify their leaves^8^. Do pitchers really represent a product of the epiascidiation process? To address this question, we performed cross-sections of the stage 1 leaf, one at the apex and the other at the middle portion of the in-rolled leaf base lamina (Fig. 2f,g). The cross-sectioned photomicrograph of the apex region reveals the presence of a hollow structure with two protruding outgrowths at one end reminiscent of the leaf base margins (Fig. 2x,y), indicating that pitcher formation occurs early in development. Cells in the apex region are differentiated into two distinct types separated by a darkly-stained layer of vascular bundles. Cells formed away from the hollow region are bigger in size and irregularly shaped while those cells facing toward the hollow region are smaller in size and rectangularly shaped. Interestingly, both cells are identical, in structure and arrangement, to those present in the leaf base lamina (magnified images in Fig. 2x,y). Thus, our findings indicated that both structures - the apex region (pitcher) and the in-rolled leaf base lamina - share anatomical features. These observations together provide evidence to the assertion by Juniper *et al*.^8^ that the modification of the *Nepenthes* leaf into a pitcher involves an epiascidiation process followed by marginal fusion. More research is needed to determine how and what drives the fusion of the *Nepenthes* leaf margins.

**Figure 3:**
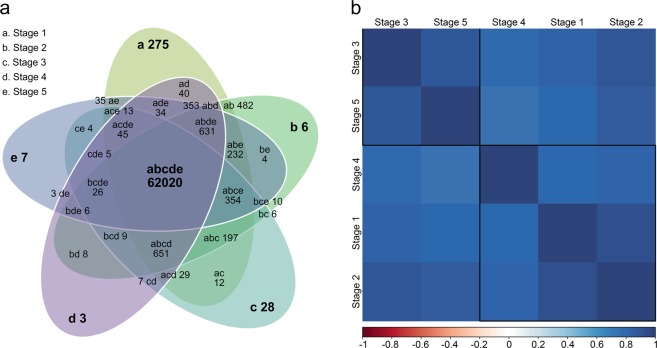
Transcript abundance estimation and correlation analysis of different stages of *N*. *khasiana* leaf development. (**a**) A Venn diagram showing the number of shared and unique transcripts among the five developmental stages of *N*. *khasiana* leaf. (**b**) The correlation of different stages based on the log_2_ FPKM values.

**Figure 4:**
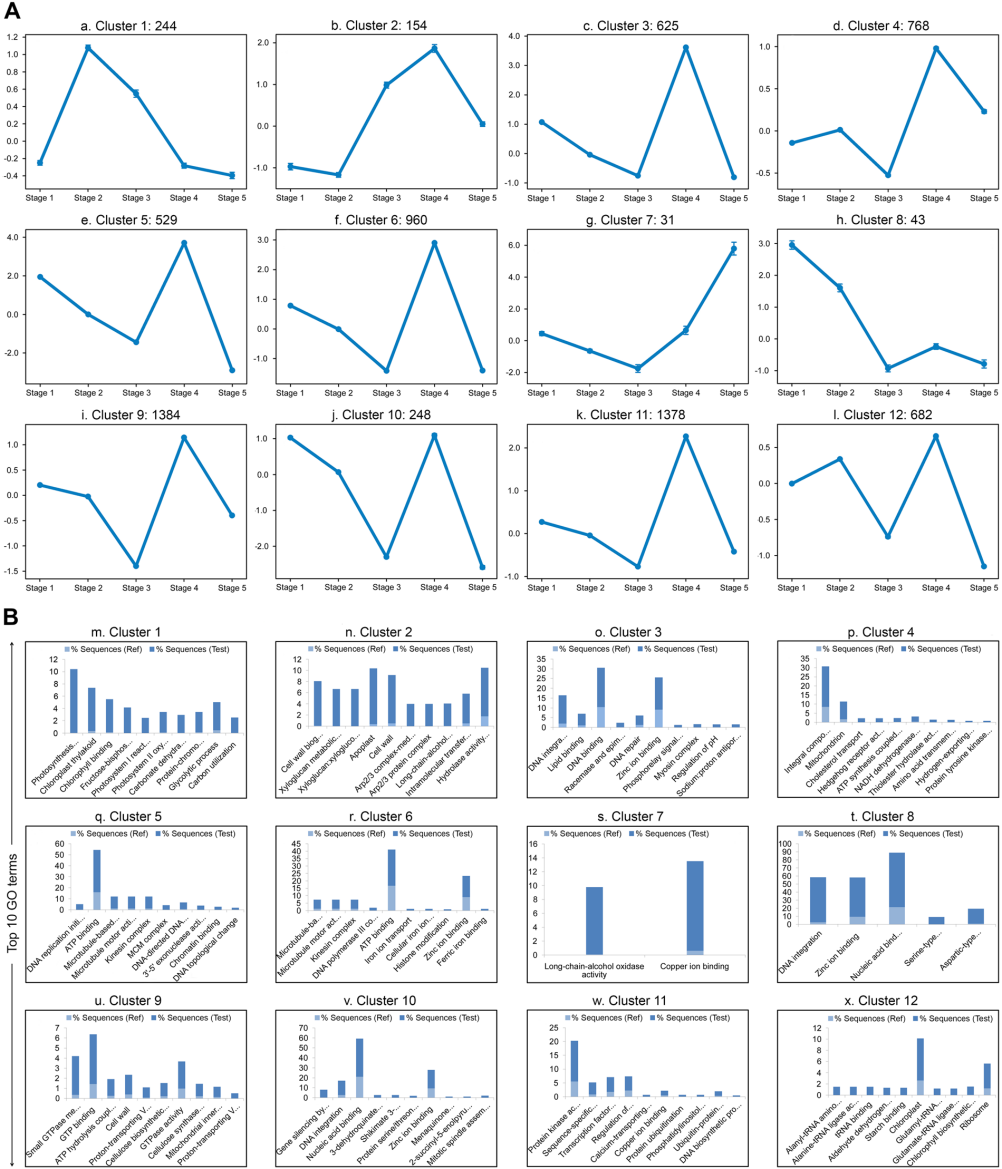
K-means clustering and functional enrichment analysis of 7047 significantly DEGs. (**A**) (**a–l**), DEGs are grouped into 12 clusters, each showing different expression patterns with relatively higher or exclusive expression at each stage of *N*. *khasiana* leaf development (numbers represent the number of DEGs for each cluster; error bars denote mean ± SE). (**B)** (**m–x**), top 10 GO terms for each cluster.

**Figure 5:**
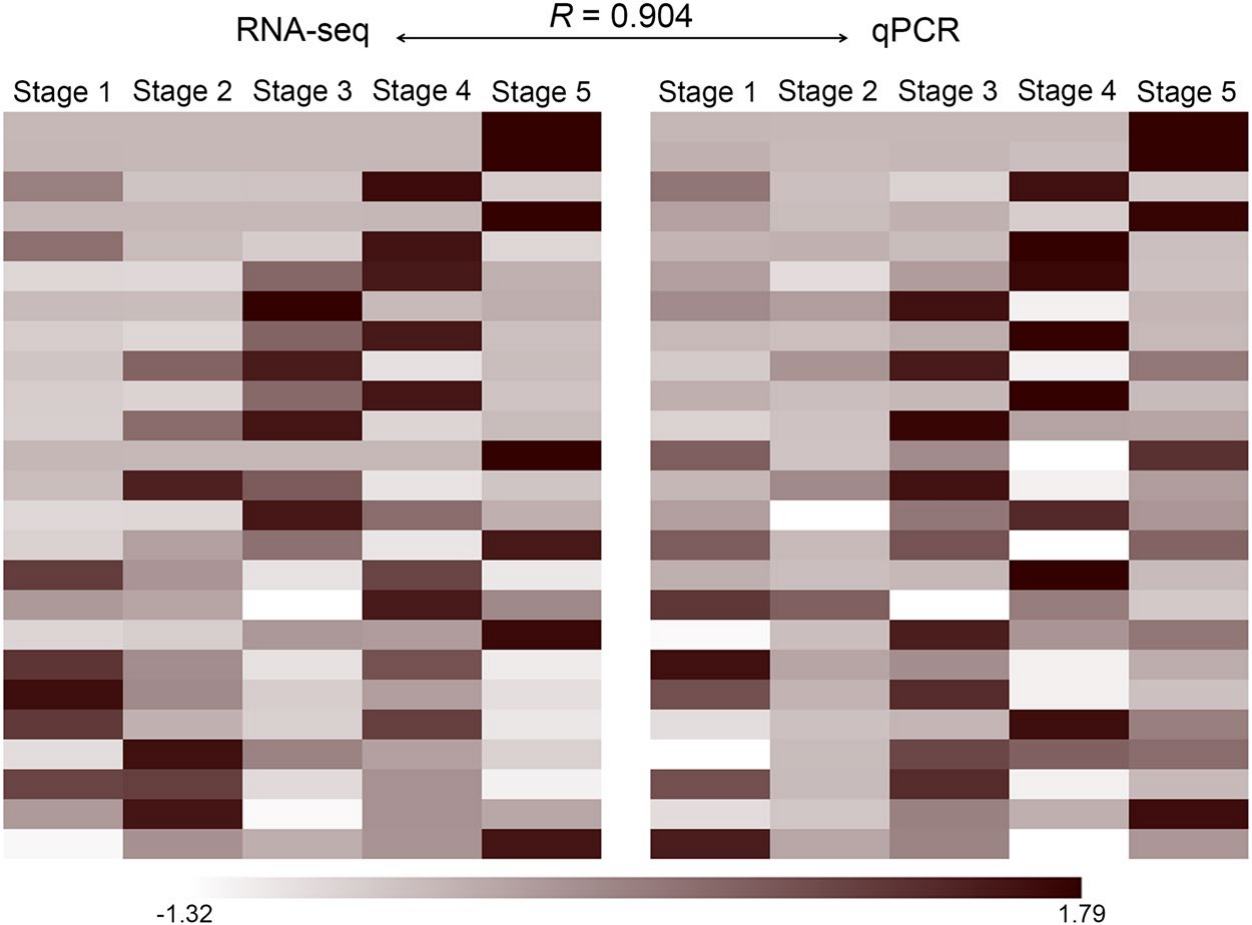
Real-time qPCR validation of RNAseq data using 25 randomly selected DEGs. *R* denotes correlation between RNA-seq and qPCR data. Colour bar represents normalized FPKM and 2^−ΔCt^ values of RNA-seq and qPCR data, respectively.

We adopted the same strategy of selecting the different stages of *N*. *khasiana* leaf development, as depicted in Fig. 2a, to investigate transcriptional changes during the development of the highly specialized *N*. *khasiana* leaf. In total, we generated around 270 million high quality cleaned paired-end reads (Table S1, Supplementary information). Reads were pooled, normalized and assembled using the freely available software Trinity. *De novo* assembly yielded 576,563 transcripts, having mean contig length of 730.89 bp and maximum contig length of 21,003 bp with an N50 length of 1,374 bp. All assembled transcripts were found to be of length more than 200 bp (Fig. S2, Supplementary information). The assembled transcripts (≥200 bp) were compared with NCBI non-redundant protein database using BLASTX program. Matches with E-value ≤ 10^−5^ and similarity score ≥ 50% were retained for further annotation. Around 60% of the transcripts found using BLASTX have confidence level of at least 1e-5, which indicated high protein conservation (Fig. S3a, Supplementary information). Close to 56% of the assembled transcripts found using BLASTX have similarity of more than 60% at protein level with the existing proteins available at the NCBI database (Fig. S3b, Supplementary information). We then aligned individual reads from each stage to the reference transcriptome to estimate transcript abundance among the five different stages of *N*. *khasiana* leaf development. On average, 93.8% of reads were properly aligned to the reference transcriptome with 308,800 unique transcripts possessing FPKM ≥ 1. Out of 308,800 unique transcripts, we identified 103,301 (33.45%) transcripts having significant BLASTX hit against the NCBI non-redundant database, of which 65,535 (21.22%) transcripts matched UniProt proteins. Out of 65,535 transcripts, 7,047 were found to be significantly differentially expressed. A total of 62,020 transcripts were commonly expressed in all five developmental stages (Fig. 3a). Among the uniquely expressed transcripts, stage 1 recorded 275 transcripts followed by stage 3 (28), stage 5 (7), stage 2 (6) and stage 4 (3). Transcripts exclusively expressed in each developmental stage are listed in Table S2, Supplementary information. From the correlation analysis of the five developmental stages of *N*. *khasiana* leaf development, stages 1 and 2 showed high correlation (Fig. 3b). This is expected as both stages are of continuous developmental processes and share morphological characteristics, except for the fact that in stage 1 the leaf base lamina is in-rolled while in stage 2, the leaf base lamina is becoming flattened (Fig. 2c,h). Surprisingly, stage 3 showed high correlation with stage 5 rather than stage 4 (Fig. 3b). The emergence of the pitcher tube becomes prominent at stage 3 while in stage 5 the pitcher is highly expanded and elongated (Fig. 2a,d). It is difficult to draw a conclusion from the correlation analysis, but as can be seen in Fig. 4, most transcripts that are highly expressed at stage 4 got reduced at both stages 3 and 5. Real-time qPCR validation of 25 randomly selected significantly differentially expressed genes (DEGs) corroborated the RNA-seq results with an overall Pearson correlation of 0.904 (Fig. 5 and Table S3, Supplementary information).

To identify genes enriched in each of the five stages of *N*. *khasiana* leaf development defined in the present study, we performed k-means clustering of the significantly DEGs using Cluster 3.0. The DEGs were grouped into 12 clusters (Fig. 4A). We then performed functional enrichment for each cluster using BLAST2GO PRO (Supplementary data S1). The top 10 GO terms for each cluster is represented in Fig. 4B. Table 1 shows a list of DEGs enriched in each cluster (top10 GO terms). A summary of the GO terms and representative genes enriched in each cluster in relation to the five different stages of *N*. *khasiana* leaf development is given in Note S1, Supplementary information.

**Table 1:** List of differentially expressed genes enriched in each cluster (top 10 GO terms).

GO ID	P value	Gene Count	DEGs	GO ID	P value	Gene Count	DEGs
***Cluster 1***				***Cluster 2***			
GO:0009765	9.43E-33	7	*LHCB1*.*2*, *LHCB1*.*4*, *CAB*, *CAB13*, *LHCB6*, etc	GO:0042546	2.68E-16	3	*XTH28*, *XTH6*, *COBL4*
GO:0009535	3.38E-15	11	*atpB*, *petC*, *NDHJ*, *psaI*, *NAD1*, etc	GO:0010411	9.66E-16	3	*XTH28*, *XTH6*, *COBL4*
GO:0016168	5.87E-14	5	*LHCB1*.*2*, *LHCB1*.*4*, *psaK*, *psbB*, *CAB*	GO:0016762	9.66E-16	3	*XTH28*, *XTH6*, *COBL4*
GO:0004332	1.22E-13	1	*FBA*	GO:0048046	1.80E-15	4	*XTH28*, *XTH6*, *LAC4*, *COBL4*
GO:0009538	1.57E-09	4	*psaE*, *psaF*, *psaL*, *psaH*	GO:0005618	1.62E-11	4	*XTH28*, *XTH6*, *LAC4*, *COBL4*
GO:0009654	3.28E-08	2	*PSBQ*, *psbR*	GO:0034314	8.79E-11	1	*ARPC2B*
GO:0004089	3.61E-08	3	*BCA1*, *CA*, hypothetical protein (HP)	GO:0005885	8.79E-11	1	*ARPC2B*
GO:0018298	5.73E-08	5	*LHCB1*.*2*, *LHCB1*.*4*, *psbB*, *CAB*, *psbB*	GO:0046577	3.72E-08	1	*FAO4A*
GO:0006096	1.81E-07	2	*FBA*, *PGI*	GO:0016866	3.41E-06	2	*BAS*, *LUP*
GO:0015976	6.27E-07	2	*BCA1*, HP	GO:0004553	6.52E-06	3	*XTH28*, *XTH6*, *CARSR12*
***Cluster 3***				***Cluster 4***			
GO:0015074	1.74E-39	8	Retrotransposon, *IN*, *Ty3/gypsy*, gag-pol, HPs	GO:0016021	1.44E-26	68	*psbD2*, *ABCB11*, *TET2*, *AQP*, *LTI6A*, etc
GO:0008289	2.48E-13	2	*REV*, *ATHB-15*	GO:0005739	8.19E-26	32	*ATP1*, *MT-ND2*, *ORF155B*, *CCMB*, *NAD4*, etc
GO:0003677	1.94E-11	50	*RPA32*, *MADS*, *REV*, *DOF*, *ZHD3*, etc	GO:0030301	6.29E-16	1	*NPC1*
GO:0016855	3.02E-09	1	*REV*	GO:0008158	6.29E-16	1	*NPC1*
GO:0006281	2.17E-08	12	*RPA32*, *ROS1*, *rpoB*, *FAN1*, *MLH1*, etc	GO:0042773	2.58E-10	6	*MT-ND2*, *NAD4*, *MT-ND4*, *MT-ND5*, *CYTB*, etc
GO:0008270	2.19E-08	22	*BAHCC1*, *RANBP2*, Retrotransposon, *IN*, gag-pol, etc	GO:0008137	9.12E-10	8	*MT-ND2*, *NAD4*, *MT-ND4*, *MT-ND5*, *MT-ND9*, etc
GO:0000160	4.90E-07	2	*APRR2*, *ETR2*, HP	GO:0016790	1.58E-09	1	*FATB*
GO:0016459	1.28E-06	1	*MYOSIN17*	GO:0015171	1.53E-07	5	*CAT2*, *At3g13620*, *CAT1*, *At1g31830*, *CAT8*
GO:0006885	1.36E-05	1	*NHX1*	GO:0008553	1.82E-07	2	*HA*, HP
GO:0015385	1.36E-05	1	*NHX1*	GO:0004713	1.82E-07	2	*At1g49730*, HP
***Cluster 5***				***Cluster 6***			
GO:0006270	1.34E-31	6	*MCM2*, *MCM6*, *CDC6*, *MCM3*, *MCM4*, etc	GO:0007018	6.66E-17	11	*KLP3*, *KIN12B*, *KIN4*, *KINUB2*, *KINUB3*, etc
GO:0005524	2.96E-31	54	*KLP3*, *ULK4*, *CDC2*, *CHR*, *atpB*, etc	GO:0003777	6.66E-17	12	*KLP3*, *KIN12B*, *KIN4*, *KINUB2*, *KINUB3*, etc
GO:0007018	1.01E-28	12	*KLP3*, *KIN1*, *NACK1*, *KIF4*, *KIN5C*, etc	GO:0005871	6.66E-17	12	*KLP3*, *KIN12B*, *KIN4*, *KINUB2*, *KINUB3*, etc
GO:0003777	1.01E-28	12	*KLP3*, *KIN1*, *NACK1*, *KIF4*, *KIN5C*, etc	GO:0009360	2.26E-16	3	*STIL*, *STIL-2*, *STIL-4*
GO:0005871	1.01E-28	12	*KLP3*, *KIN1*, *NACK1*, *KIF4*, *KIN5C*, etc	GO:0005524	1.79E-08	85	*CIPK21*, *At1g60630*, *KLP3*, *CHR*, *At5g41260*, etc
GO:0042555	7.74E-26	5	*MCM2*, *MCM6*, *MCM3*, *MCM4*, HP	GO:0006826	4.07E-07	2	*FER3*, *FER*
GO:0003887	2.64E-16	4	*POLE2*, *POLA*, *POLA2*, *POLE1*	GO:0006879	4.07E-07	2	*FER3*, *FER*
GO:0008408	2.87E-13	2	*POLA*, *POLE1*	GO:0016570	9.14E-07	2	At5g08430, *ADA2*
GO:0003682	2.97E-12	2	*CMT3*, HP	GO:0008270	1.05E-06	48	*SIZ1*, *BAHCC1*, *RNF34*, *DRIP2*, *PAT18*, etc
GO:0006265	4.30E-12	3	*TOP2*, *TOP3A*, HP	GO:0008199	3.30E-06	2	*FER3*, *FER*
***Cluster 7***				***Cluster 8***			
GO:0046577	1.27E-05	1	*FAO4A*	GO:0015074	9.36E-26	4	Retrotransposon, *Ty3/gypsy*, *IN*, HP
GO:0005507	5.36E-05	1	*AO*	GO:0008270	6.10E-11	2	Retrotransposon, *GATA15*, *Ty3/gypsy*, *IN*, HP
				GO:0003676	1.52E-10	7	*GATA15*, Ty3/gypsy, IN, Retrotransposon, *ERF3*, etc
				GO:0004867	1.24E-09	2	HPs
				GO:0004190	5.78E-09	1	HP
***Cluster 9***				***Cluster 10***			
GO:0007264	1.07E-22	14	*ARF8*, *RABA2A*, *RAC5*, *RAC13*, *ARF*, etc	GO:0031047	2.46E-16	1	HP
GO:0005525	2.23E-13	20	*ARF8*, *TUBA6*, *RABA2A*, *RAC5*, *RAC13*, etc	GO:0015074	3.50E-15	7	*Ty3/gypsy*, Retrotransposon, *IN*, HPs
GO:0015991	1.67E-08	9	*VHA-H*, *VHA-B*, *VHA-D2*, *VAT-M*, *VHA-A3*, etc	GO:0003676	1.24E-09	24	*MYB23*, *MYB1*, *BZIP34*, *H3*.*2*, *RBG2*, etc
GO:0005618	3.63E-08	4	*EXP11*, *SBT*, *EXP9*, *XTH8*	GO:0003855	2.19E-09	1	*EMB3004*
GO:0033179	1.67E-07	4	*VHA-D2*, *VAT-M*, *VHA-A3*, *VHA-C*	GO:0004764	2.19E-09	1	*EMB3004*
GO:0030244	1.32E-06	3	*CESA1*, *CESA2*, *CESA3*	GO:0004712	1.07E-06	2	*MPS1*, *MPH1*
GO:0003924	3.94E-06	15	*TUBA6*, *RABA2A*, *RABE1C*, *GP*, *RABA5D*, etc	GO:0008270	1.21E-05	4	*IN*, Retrotransposon, HPs
GO:0016760	4.11E-06	3	*CESA1*, *CESA2*, *CESA3*	GO:0009234	4.40E-05	2	*PHYLLO*, HP
GO:0005743	7.83E-06	8	*MPC2*, *AAC*, *CYTB8*, *atpC*, *MT-ND2*, etc	GO:0070204	4.40E-05	2	*PHYLLO*, HP
GO:0033180	1.12E-05	4	*VHA-H*, *VHA-B*, *VHA-C*, HP	GO:0007094	1.21E-04	1	*BUBR1*
***Cluster 11***				***Cluster 12***			
GO:0004672	3.51E-27	60	*CPK1*, *At2g20050*, *IRE1A*, *At5g57670*, *At1g80640*, etc	GO:0006419	6.87E-11	1	*EMB1030*
GO:0043565	8.16E-16	13	*GATA26*, *ANL2*, *TGA2*, *BZIP*, *AREB*, etc	GO:0004813	6.87E-11	1	*EMB1030*
GO:0003700	5.35E-12	20	*NFXL1*, *SEP2*, *GATA8*, *EIN3*, *TGA2*, etc	GO:0000049	6.87E-11	1	*OVA3*
GO:0006355	6.09E-08	21	*CYCT1–4*, *NAC7*, *GATA8*, *ARF9*, *CUC*, etc	GO:0004030	7.18E-10	1	*FALDH*
GO:0005388	8.36E-08	3	*ACA10*, *ACA9*, *ECA3*	GO:2001070	7.18E-10	2	*SS3*, *FKFBP*
GO:0005507	4.98E-07	4	LAC14, LAC4, RAN1, HMA5	GO:0009507	8.36E-10	28	*PRXQ*, *YCF4*, *RPC2*, *rpoC*, *rpoA*, etc
GO:0016567	7.57E-07	1	*RCHY1*	GO:0006424	7.49E-09	1	*OVA3*
GO:0016307	7.57E-07	2	*FAB1B*, HP	GO:0004818	7.49E-09	1	*OVA3*
GO:0004842	3.57E-06	5	*ARI7*, *PUB1*, *ARI8*, *PUB33*, HP	GO:0015995	1.50E-08	3	*CHLG*, *CHLD*, *CRD1*
GO:0071897	1.12E-05	2	*LIG4*, *LIG1*	GO:0005840	4.79E-08	19	*RPL1*, *RPL27*, *RPL20*, *RPS10*, *RPL15*, etc

A complete list of all DEGs can be found in Supplementary data S1; HP includes hypothetical or uncharacterized proteins.

**Figure 6:**
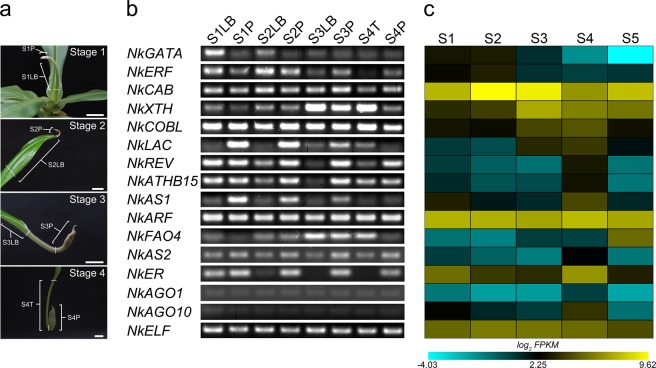
Determination of the local expression of selected genes in the pitcher part of the first four stages of *N*. *khasiana* leaf development. (**a**) Illustrations depicting the dissection of tissues for RT-PCR analysis of selected enriched and/or related genes from the first four developmental stages viz. leaf base (S1LB) and pitcher (S1P) of stage 1, leaf base (S2LB) and pitcher (S2P) of stage 2, leaf base (S3LB) and pitcher (S3P) of stage 3 as well as the tendril (S4T) and pitcher (S4P) of stage 4 (white vertical/horizontal lines specify the dissected regions; bar = 1 cm). (**b)** Expression analysis of selected genes in the dissected tissues of the first four developmental stages by RT-PCR. Cropped gels photos separated from each other by white space are provided here for clarity and conciseness. Full-length gels photos of each gene are represented in Fig. S8, Supplementary information. (**c**) Heatmap showing log_2_ FPKM values of selected genes at different stages of development. S1 - stage 1, S2 - stage 2, S3 - stage 3, S4 - stage 4, and S5 - stage 5.

To identify which of these enriched genes play a role in the development of *Nepenthes* pitcher, we further dissected the first four developmental stages into different parts to separate the pitcher from the leaf base or tendril (Fig. 6a) and performed RT-PCR to check for their localized expressions (Fig. 6b). We also performed qPCR to accurately quantity the expression of selected genes. The RT-PCR results show two genes, *NkLAC* and *NkAS1*, which are expressed exclusively or at higher levels in the pitcher of stages 1–3 (Fig. 6b). In Arabidopsis, *LAC* is required for lignin polymerization^14^, and studies have reported the presence of lignin in Arabidopsis trichome cell walls^15^. Therefore, enhanced expression of *NkLAC* in the pitcher may be associated with lignin synthesis in the trichomes covering the apex region (Fig. 2f,i,l). The *AS1* gene is expressed during initiation of leaf primordia and acts to exclude KNOX proteins from initiating leaves^16^. Overexpression of *AS1* in Arabidopsis Col background causes plants to develop narrower leaves and longer petioles^17^; in L*er* background, however, plants showed stunted growth but developed normal leaves^18^. When *AS1* is mutated in the Arabidopsis L*er* background, leaf initiation persists; but in severe cases, the first two rosette leaves displayed novel leaf phenotype typical of a lotus leaf, in which the petiole is attached to the abaxial surface of the leaf lamina^18^. Interestingly, flattened leaves of the L*er*-background *as1* mutant lack a prominent midvein. Similar phenotypes were also observed in Arabidopsis *as*2 mutants, implying that both AS1 and AS2 function in the same regulatory pathway by forming protein complexes, which in turn act to regulate downstream genes during leaf development^18^. The AS1-AS2 complexes are localized in the adaxial domain and together with ER promote leaf polarity establishment^18^. To specify the role of *AS1* in *Nepenthes* pitcher formation, we examined the expressions of *AS2* and *ER* homologs in *N*. *khasiana*. The results show that *NkAS2* is expressed in all the dissected tissues with relatively higher expression in the pitcher (Fig. 6b). *NkER*, on the other hand, displayed exclusive expression in the pitcher of stages 2–4 with almost same levels of expression in stage 1 (Fig. 6b). The qPCR results for *NkAS1*, *NkAS2* and *NkER* match the results seen in the RT-PCR analysis, with a few exceptions (Fig. S4, Supplementary information). These exceptions include *NkAS2* expression in the dissected tissues of stage 4 (higher in tendril) and *NkER* expression in the dissected tissues of stage 1 (higher in pitcher). Thus, it is safe to say that higher *AS1* expression is correlated with an increased expression of *NkAS2* and *NkER* in the pitcher of *N*. *khasiana*. But based on the information available for Arabidopsis, it is unlikely that *AS1* may play a direct role in the formation of the *N*. *khasiana* pitcher.

Further, we show that the expression of *NkREV* was higher in the pitcher than the leaf base lamina in stages 2–4, with similar levels of expression in the two dissected tissues of stage 1 (Fig. 6b). Similar expression pattern was also observed for *NkATHB15*; but in the tendril and pitcher of stage 4, *NkATHB15* expressions were of the same levels (Fig. 6b). Although the expression of *NkATHB15* is of interest, overexpressing *ATHB15* in Arabidopsis, however, do not show any phenotype^19^. In Arabidopsis, reports have shown that a single nucleotide change in the putative lipid/sterol-binding START domain of the *REV* gene results in gain-of-function mutation^20,21^. This gain-of-function mutation causes leaves to become trumpet-shaped. In severe cases, these trumpet-shaped leaves grew out from the abaxial midvein^21^. Unlike gain-of-function mutations in *PHB* and *PHAVOLUTA* (*PHV*)^22^, the inside surface of the trumpet-shaped leaf in gain-of-function *rev* mutants is adaxial while the outside surface is abaxial, similar to the *Nepenthes* pitcher. The *rev* phenotype is caused as a result of the failure on the part of miR165/166 to regulate *REV*^20^. Failure in miRNA regulation may be due to a reduced level of *AGO1*, which is known to direct miRNAs to target sequences^23^. Alternatively, *AGO10* may compete with *AGO1* for miR165/166 to prevent post-transcriptional modification of the *REV* transcript^24^. In light of these findings, we performed RT-PCR to determine the expression patterns of *Nepenthes* homologs of *AGO1* and *AGO10*. Contrary to our expectations, the results show reduced levels of expressions for both genes, in comparison to *NkREV* expressions, across all the dissected samples (Fig. 6b). To test whether reduced levels of *NkAGO1* and *NkAGO10* expressions is a result of primer failure to amplify the targeted genes, we increased the cycles of PCR amplification and found that *NkAGO1* expression levels increases but remained uniform throughout the different dissected tissues whereas *NkAGO10* displayed relatively higher expression in the pitcher parts of stages 2–4 (Fig. S5, Supplementary information). The qPCR results for *NkREV* and *NkATHB15* expressions across the dissected tissues corroborated the RT-PCR results (Fig. S6, Supplementary information). Barring a few exceptions (higher expression in the pitcher of stage 1 and similar expression in both tissues of stage 4), the qPCR result for *NkAGO10* matches the one conducted at increased cycles of RT-PCR amplification (35 PCR cycles). For *NkAGO1*, the qPCR result indicated higher level of expression in the pitcher than leaf base of stages 1–4, which contradicts with the RT-PCR result (Fig. S6, Supplementary information). It is likely then that other regulatory mechanisms, other than *AGO1*/*AGO10*, may be controlling the expression of *REV* in the developing pitchers of *N*. *khasiana*.

## Conclusion

In *Insectivorous Plants*, Darwin imagined that the origin of carnivory in angiosperms is a result of natural selection acting on sticky gland-bearing plants that are then adapted over time to digest the captured prey^25^. This proposition raises the question of what strong selection pressure could have directed the evolution of such extraordinary events of morphological innovation. We now know that the origin of megaphyllous leaves in land plants is linked with a 90% reduction in atmospheric CO_2_ and the concurrent increase in stomatal densities to prevent overheating^26^. It can be assumed then that the nutrient-poor habitat in which carnivorous plants grow might have imposed strong selective pressure resulting in the evolution of innovative leaf morphologies. In the event of such an adaptive transformation, recruitment of common developmental programmes evident in typical angiosperm leaves occurred but is altered in a way to generate morphological novelty. In the present study, we identified a number of candidate genes that might play a role in the development of the *Nepenthes* pitcher. Of these, *AS1* and *REV* are of significant interest as evidenced by higher *NkAS1* and *NkREV* expressions in the developing pitchers of *N*. *khasiana*. How these genes are recruited to modify a *N*. *khasiana* leaf into a pitcher require further investigation. This involves the use of RNA *in situ* hybridization techniques, functional validation of candidate genes in Arabidopsis mutants and/or silencing of the candidate genes in *N*. *khasiana* by RNA interference.

## Methods

### Plant material and growth conditions

*Nepenthes khasiana* plants were collected from their natural habitat located at Jaraiñ, Jaiñtia Hills District, Meghalaya (25° 18.651″ N, 92° 07.786″ E), transferred into pots containing Soilrite Mix (Keltech Energies Ltd, Bangalore) and kept at the greenhouse of the School of Life Sciences, Jawaharlal Nehru University, New Delhi with temperature and humidity maintained at 28 ± 2 °C and 60 ± 5%, respectively.

### Examining growth and development of the *N*. *khasiana* leaf

We examined growth and development of the *N*. *khasiana* leaf from the initial stages of development visible to the naked eye (Fig. 1). Following the examination, five stages of *N*. *khasiana* leaf development were selected for RNA-seq (Fig. 2a–e). This selection is based on the observation that at a particular period in the growth and development of young *N*. *khasiana* plants, five distinct stages of leaf development can be seen in different developing leaves (Fig. 2a). The topmost slender leaf (L1) was considered stage 1 while the leaf (L2) with the expanding leaf base was considered stage 2 (Fig. 2a–c). The third leaf from the top (L3), showing complete expansion of the leaf base and the emergence of the pitcher tube, characterized stage 3 (Fig. 2a,d). The leaf (L4) showing elongation of the tendril and expansion of the pitcher tube represented stage 4 (Fig. 2a,e). The leaf (L5) with the expanded unopened pitcher was considered stage 5 (Fig. 2a).

### Scanning electron microscopy (SEM)

Tissues from each stage were cut into smaller pieces and fixed in 0.1 M phosphate buffer (0.1 M Na_2_HPO4, pH adjusted to 7.2 by adding 0.1 M NaH_2_PO4) containing 2.5% glutaraldehyde. As depicted in Fig. 2a–e, these tissue pieces represent portions of the leaf base lamina as well as the tip of the developing leaf of stages 1–3. The developing pitchers of stages 4 and 5 were cut into separate pieces at three different locations - top, a little above the middle and bottom - which correspond to the lid, waxy zone and digestive zone, respectively. In addition, the leaf base lamina midvein and the tendril of the later stages of *N*. *khasiana* leaf development (stages 3, 4 and 5) were also cut into smaller pieces and fixed in the aforesaid fixative. Fixed samples were processed for SEM at the Advanced Instrumentation Research Facility, Jawaharlal Nehru University, New Delhi and viewed under JEOL JSM-6360 SEM.

### Histological analysis

Tissues from stage 1 were cut into two separate pieces: one representing the apex and another from the middle portion of the leaf base lamina (Fig. 2f,g), and fixed in 2.5% glutaraldehyde in 0.1 M phosphate buffer (pH 7.2). Fixed tissue samples were processed, cross-sectioned, stained with 0.05% toluidine blue, viewed and photographed using a Nikon Eclipse Ti-S Inverted Microscope.

### RNA extraction, library preparation and sequencing

For RNA extraction, tissues from each of the five different stages of *N*. *khasiana* leaf development defined in the present study were dissected using a sterile scalpel blade. As depicted in Fig. 2a, leaves of stages 1 and 2 were dissected at the base of each leaf. On the other hand, leaves of stages 3, 4 and 5 were dissected near the tip (Fig. 2a). Thus, a portion of the leaf base, tendril and pitcher make up stage 3 whereas stage 4 comprises the tendril and pitcher. Stage 5 represents the unopened pitcher (Fig. 2a). Total RNA from these dissected tissues was then isolated using the Raflex Kit (Bangalore Genei, India) and/or the Spectrum Plant Total RNA Kit (Sigma, USA), following the manufacturer’s instructions. Libraries were prepared using the TruSeq stranded total RNA library preparation kit (Illumina). Generated libraries were validated on the Agilent 2100 Bioanalyzer and sequenced using the Illumina HiSeq. 2000 platform following the manufacturer’s recommended protocol to generate 2 × 100 bp paired-end data. The RNA-seq data from two biological replicates has been submitted to NCBI short read archive and can be accessed under accession number SRR4340048. Two biological replicates represent two separate individual plants, from which tissue samples comprising the different stages were harvested and sequenced separately.

### Data pre-processing and de-contamination

Illumina adapter sequences were removed from the data using Cutadapt v1.3^27^. Low-quality data (Q < 20) were filtered using Sickle v1.33^28^. From the trimmed paired-end reads, unwanted sequences which include mitochondrial genome sequence, ribosomal RNAs, transfer RNAs, adapter sequences and non-polyA tailed RNAs were removed. The decontamination step was performed using Bowtie2 version 2.2.2^29^.

### *De novo* transcriptome assembly, annotation and differential expression analysis

Cleaned reads were pooled, normalized and *de novo* assembled using Trinity (release 20140717)^30,31^. For gene expression estimation, reads were aligned to the assembled reference transcriptome using Bowtie2 version 2.2.2. Up to 1-mismatches in the seed region (length = 31 bp) were allowed and all multiple mapped position were reported. About 93% of reads on average were properly aligned to the assembled reference transcriptome. Fragments per Kilobase of transcript per Million mapped reads (FPKM) values were calculated using SciGenom Labs Pvt. Ltd Perl script. Annotation of the assembled transcript was carried out using CANoPI (Contig Annotator Pipeline), a SciGenom Labs Pvt. Ltd pipeline. Transcripts having FPKM ≥ 1 and minimum length ≥ 200 were selected for annotation. The assembled transcripts were annotated against NCBI non-redundant protein database using BLASTX 2.2.28 program^32^ with E-value and similarity score cut-off of ≤ 10^−5^ and ≥ 50%, respectively. Using BLASTX hits, transcripts were also annotated against UniProt database. Differential gene expression analysis was performed for transcripts having read count ≥ 1 using DESeq. 3.2.0^33^, in pairwise combinations between stages - for example stage 1 vs. stage 2, stage 2 vs. stage 1, stage 1 vs. stage 3, and so on - to identify upregulated and downregulated genes in each stage. Transcripts with p-value ≤ 0.05 and read count ≥ 100 were considered significantly differentially expressed. The assembled transcript sequences have been deposited at DDBJ/EMBL/GenBank as a Transcriptome Shotgun Assembly project under the accession GFDV00000000.

### Correlation analysis

The correlation analysis among the five different developmental stages was performed using the freely available R software (https://www.r-project.org/) and the result was plotted using the ‘corrplot’ package. Prior to running the correlation analysis, FPKM values of each transcript were log_2_ transformed.

### K-means clustering analysis

Prior to clustering, we estimated the number of clusters k using R, employing the gap statistic algorithm^34^. After estimating k (k = 12, see Fig. S7, Supplementary information), we then performed k-means clustering of the significantly differentially expressed genes (DEGs, log_2_ transformed Read Counts) using Cluster 3.0^35^, applying the Euclidean distance similarity metric. We used Java TreeView program to visualize the data^36^.

### Functional enrichment analysis

Functional enrichment analysis was performed using BLAST2GOPRO 3.3^37^. Here, the annotation file comprises the list of significantly DEGs with their GO terms and gene descriptions. We then prepared separately the ‘test-set’ comprising the different clusters (1–12) generated using Cluster 3.0 and the ‘reference-set’ comprising the list of DEGs without the GO terms and gene description. Enrichment analysis was performed on all clusters separately using default settings and applying the ‘reduce to most specific’ option.

### Validation of RNA-seq data using real-time quantitative PCR (qPCR)

Real-time qPCR was used to validate the RNA-seq results of 25 randomly selected genes. cDNAs of the two biological replicates used for RNA-seq were synthesized using the First Strand cDNA synthesis kit (Thermo Scientific). Three house-keeping genes viz. Ubiquitin (*UBQ*), Actin and Elongation Factor (*ELF*) were initially tested for stable expression, out of which two (*UBQ* and *ELF*) were used to normalize expression of the selected genes. We performed qPCR experiments in a MicroAmp FAST 96-well reaction plate using a 7500 FAST Real-Time PCR System (Applied Biosystems). Each well contained 10 × diluted cDNA, 1 × Power SYBR® Green PCR Master Mix and 0.5 µM of each gene-specific primer. The PCR cycling condition is as follows: 20 sec at 50 °C, 10 min at 95 °C and 40 cycles of 15 sec at 95 °C, 1 min at 58 °C and 15 sec at 72 °C. Two biological and two technical replicates were analyzed for each sample and data analysis was performed using 7500 Software v 2.0.5. We generated the 2^−ΔCt^ values, compared them with the RNA-seq derived FKPM values and calculated Pearson Correlation (R) to check for correlation between the RNA-seq and qPCR data. qPCR primers are listed in Table S4, Supplementary information.

### Reverse transcription PCR (RT-PCR) confirmation of local enrichment of transcripts in the pitcher part of the *N*. *khasiana* leaf

We used RT-PCR to determine the localized expression of enriched genes in the dissected tissues of the first four developmental stages viz. leaf base (S1LB) and pitcher (S1P) of stage 1, leaf base (S2LB) and pitcher (S2P) of stage 2, leaf base (S3LB) and pitcher (S3P) of stage 3 as well as the tendril (S4T) and pitcher (S4P) of stage 4. The dissection scheme is illustrated in Fig. 6a. Dissected tissues of the leaf base and pitcher of stage 1 were pooled from two separate plants. We also examined the expression of other genes, available from our transcriptome data, linked either directly or indirectly to the enriched genes and/or are related to pitcher development. The *NkELF* gene was used as an internal control. Extraction of total RNA and cDNA synthesis from these dissected tissues were performed as mentioned above. Transcript-specific RT-PCR primers are listed in Table S5, Supplementary information. Amplifications were carried out in a 15 µl PCR reaction, each containing 10 × diluted cDNA, 0.5 µM transcript-specific primer, 0.25 µM dNTPs, 1.3 × Taq buffer containing MgCl_2_, 0.06 units Taq DNA polymerase and an appropriate volume of sterilized Millipore water. DNA amplification was performed in an Applied Biosystems Proflex PCR System programmed for 32 cycles (1 cycle for 5 min at 95 °C, 30 cycles for 1 min at 95 °C, 30 sec at 58 °C and 30 sec at 72 °C, followed by an additional incubation for 7 min at 72 °C for the 32nd cycle). For increased cycles of PCR amplification, the PCR system was programmed for 37 cycles. The RT-PCR products were resolved on 1.5% EtBr-stained agarose gel. Inferences about higher or reduced levels of expression were made in two ways: i) the expression of the gene under consideration in the pitcher relative to its expression in the leaf base of each stage (e.g., *NkLAC*), ii) the expression of the gene under consideration relative to the expression of an enriched gene in the dissected tissues (leaf base and pitcher) of each stage (e.g. *NkAGO1*). The RT-PCR results were also confirmed using real-time qPCR analysis for a selected number of enriched and related genes viz. *NkREV*, *NkATHB15*, *NkAS1*, *NkAS2*, *NkER*, *NkAGO1* and *NkAGO10*. Primers used are listed in Table S5, Supplementary information and qPCR was performed as mentioned above. In this case, *ELF* was used to normalize the expressions of the selected genes. The 2^−ΔCt^ values were generated for each gene and plotted using GraphPad Prism.

## Supplementary information

Supplementary information

Supplementary Data S1
