# Supplementary material for: ASYMMETRIC LEAVES1 and REVOLUTA are the key regulatory genes associated with pitcher development in Nepenthes khasiana
Source: Sci Rep. 2019 Apr 19;9:6318. doi: 10.1038/s41598-019-42779-6 (PMC6474907; doi:10.1038/s41598-019-42779-6)
Supplement: Supplementary file 1 — Supplementary information [file 41598_2019_42779_MOESM1_ESM.pdf]

Supplementary information

ASYMMETRIC LEAVES1 and _REVOLUTA_ are the key regulatory genes associated with pitcher development in _Nepenthes khasiana_

Jeremy Dkhar* and Ashwani Pareek
Stress Physiology and Molecular Biology Laboratory, School of Life Sciences, Jawaharlal Nehru University, New Delhi 110067

Email IDs: jeremydkhar@gmail.com; ashwanip@mail.jnu.ac.in

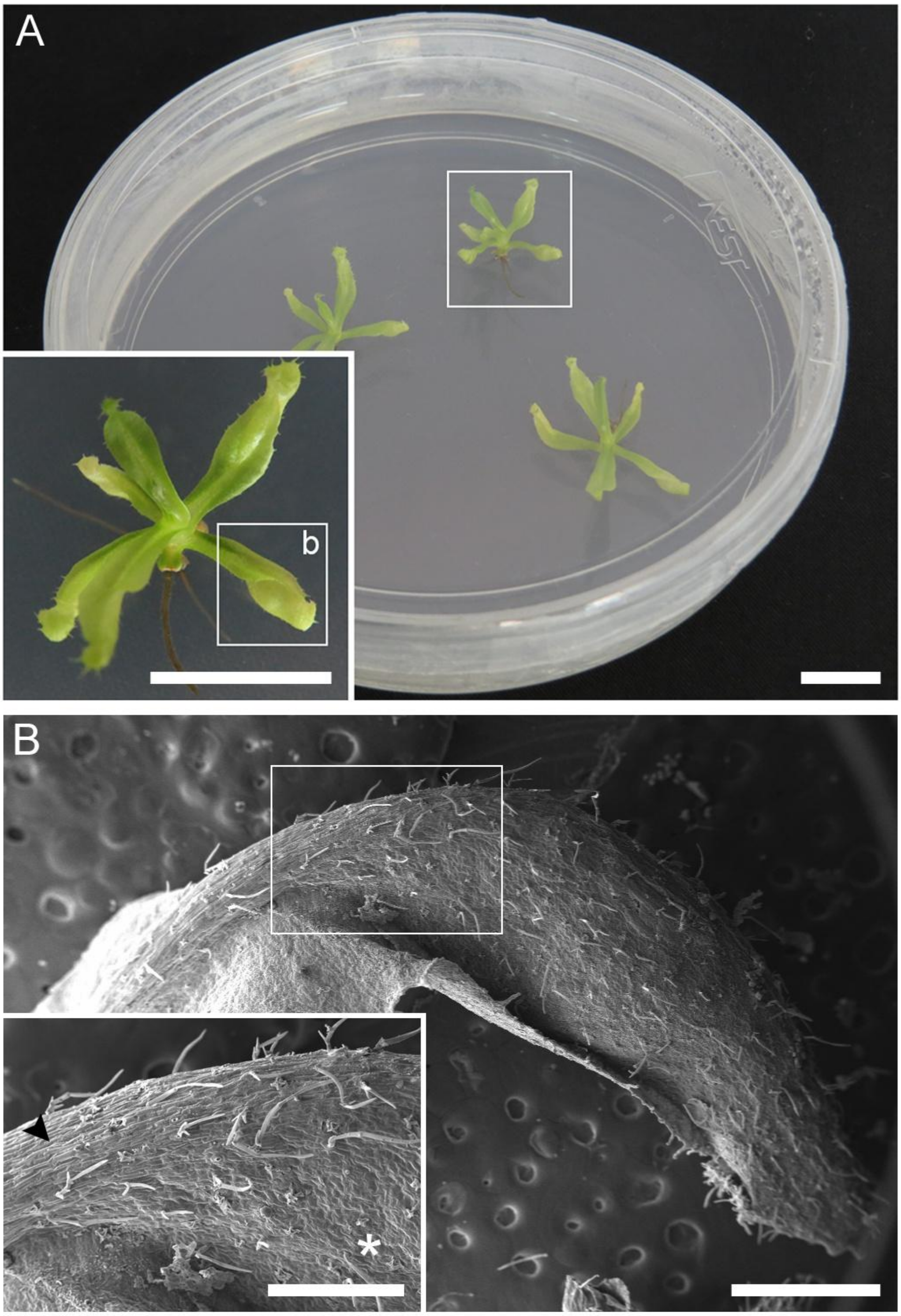

Fig. S1. **A**, *In vitro* raised *N. khasiana* plantlets. Inset shows a close-up image of one of the plantlet (box). A single leaf (**b**) was dissected and examined under a scanning electron microscope. Bar=1cm. **B**, SEM images of the dissected leaf. Inset shows magnified image of a portion of the dissected leaf (box). Here, epidermal cells lining up the midvein (black arrowhead) are clearly distinct from cells making up the epidermal surface of the pitcher (white asterisk). Interestingly, the tendril is not formed. Bar=1cm; 0.5cm (inset).

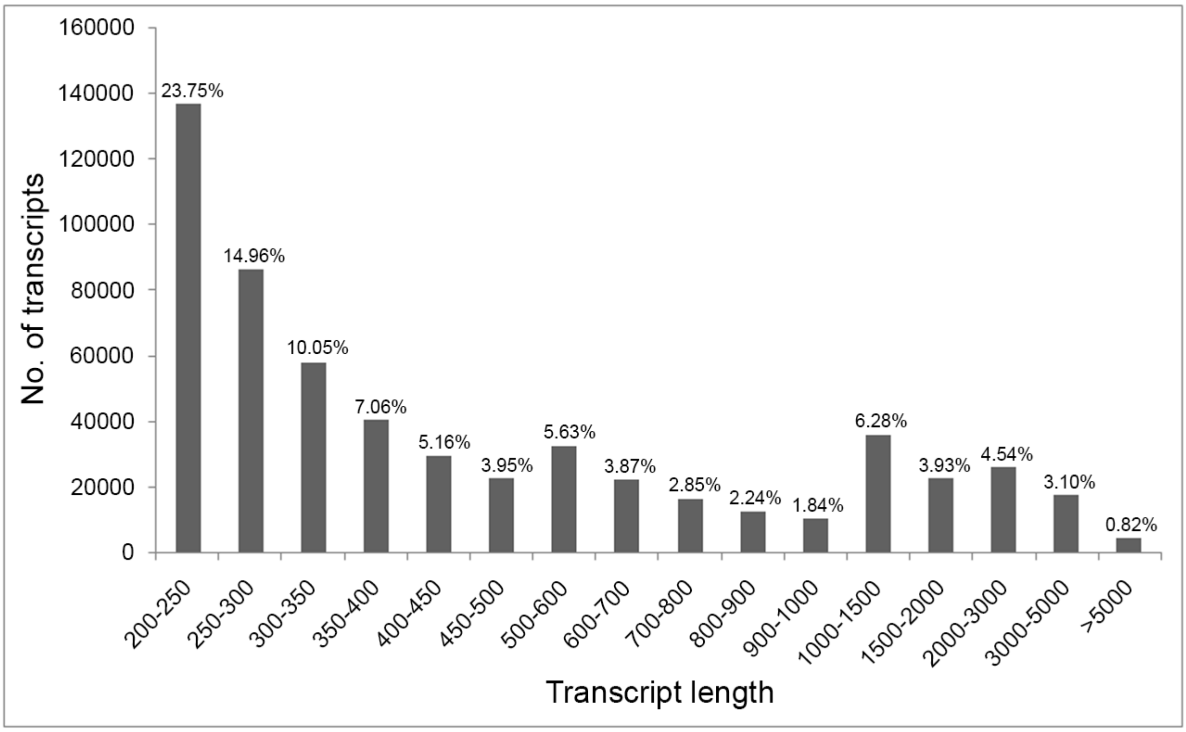

Fig. S2. Length distribution of assembled transcripts.

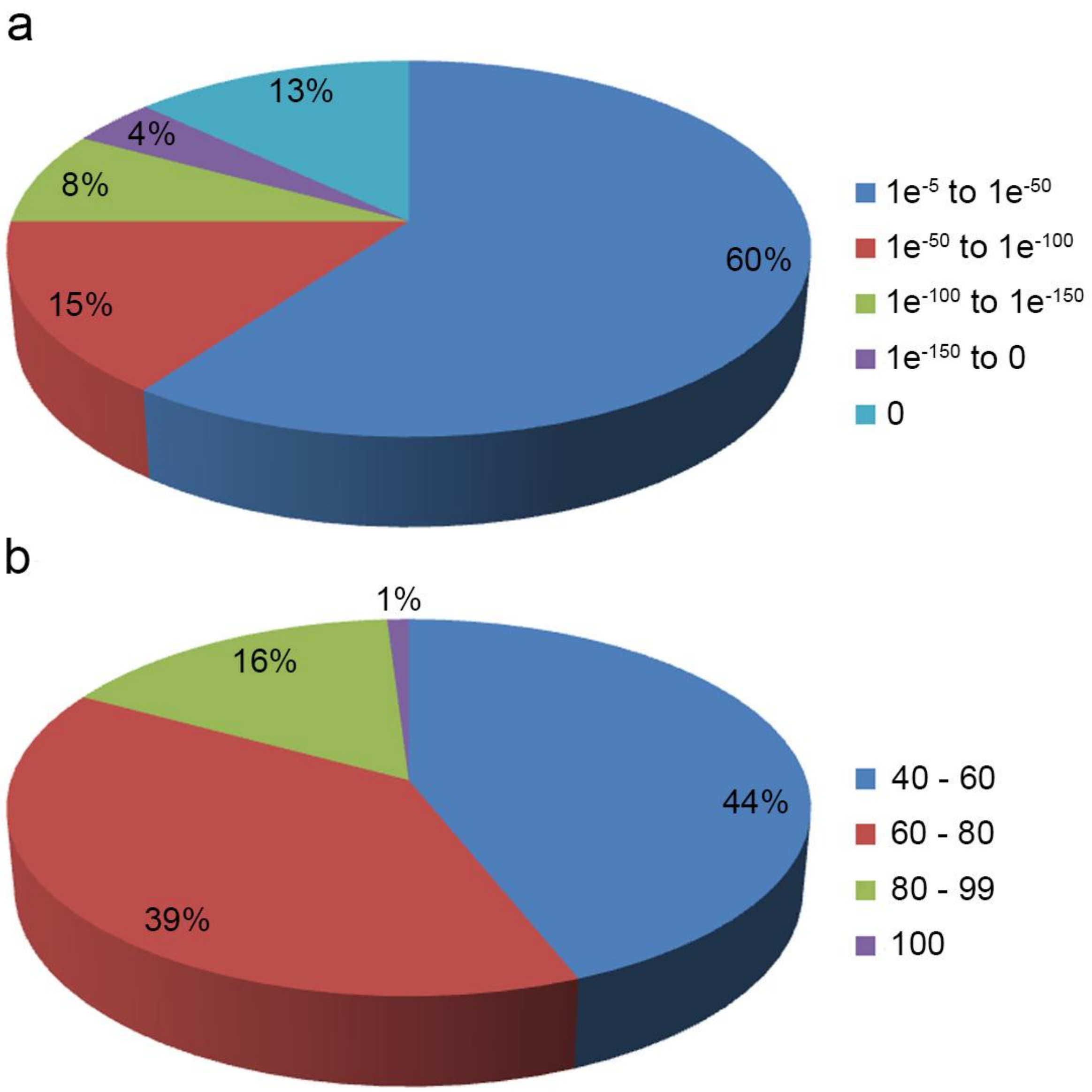

Fig. S3. **a**, BLASTX e-value distribution of transcriptome. **b**, BLASTX similarity score distribution of transcriptome.

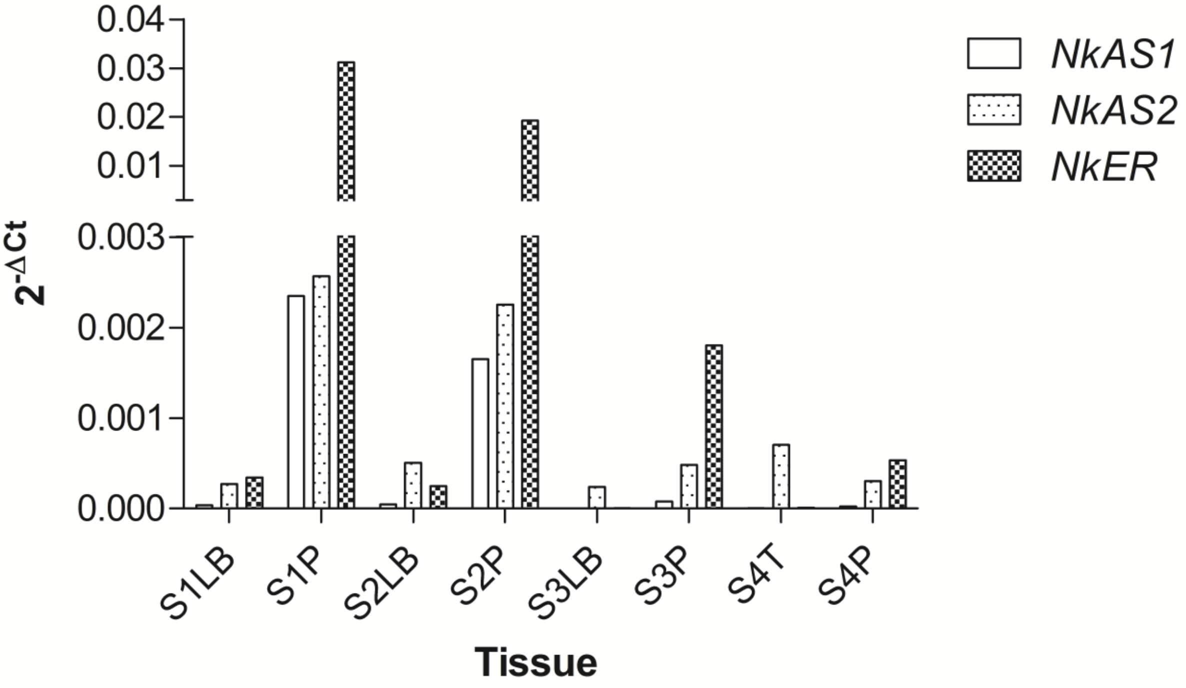

Fig. S4. Real-time qPCR analysis of *NkAS1*, *NkAS2* and *NkER* in the dissected tissues of the different stages of *N. khasiana* leaf development. The $2^{-\Delta Ct}$ values were generated for each gene and plotted against the dissected tissues.

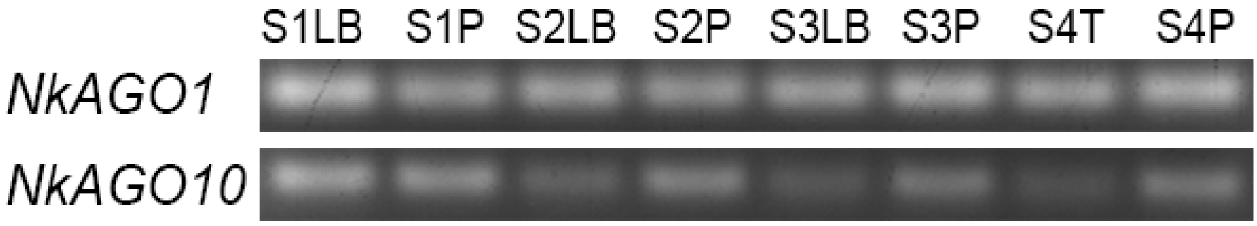

Fig. S5. RT-PCR amplification of *NkAGO1 and NkAGO10* genes at increased PCR cycles (35 cycles). Amplification was programmed as mentioned in the Methods section. Cropped gels photos separated from each other by white space are provided here for clarity and conciseness. Full-length gels photos are represented in Fig. S9 below. S1LB - leaf base of stage 1; S1P - pitcher of stage 1; S2LB - leaf base of stage 2; S2P - pitcher of stage 2; S3LB - leaf base of stage 3; S3P - pitcher of stage 3; S4T - tendril of stage 4; S4P - pitcher of stage 4.

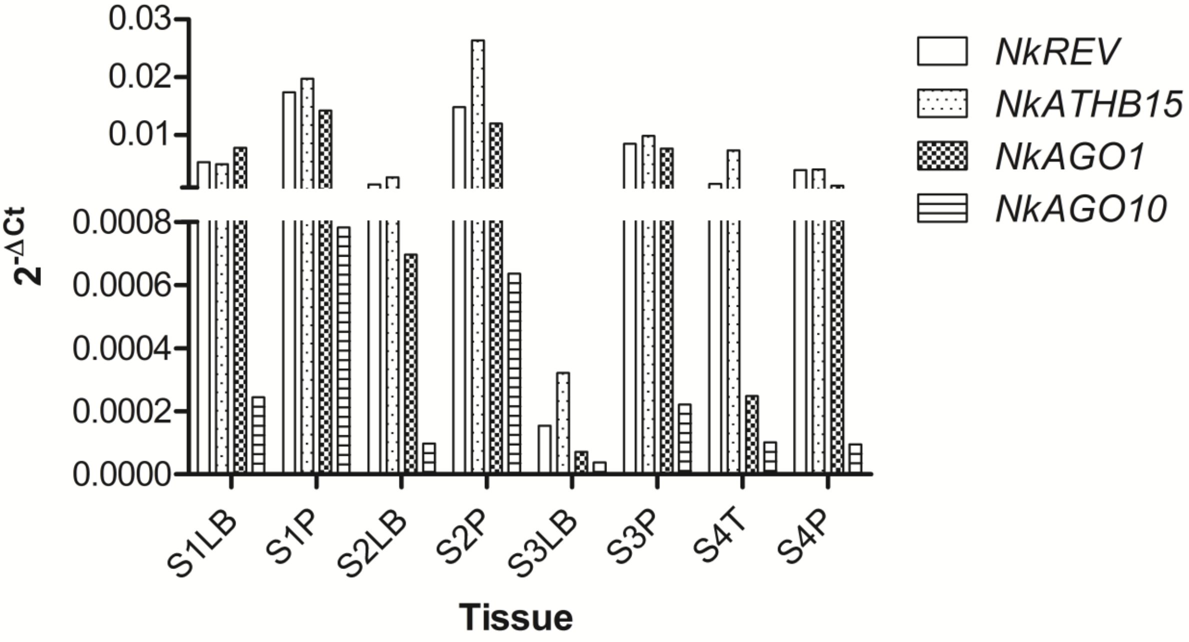

Fig. S6. Real-time qPCR analysis of *NkREV*, *NkATHB15*, *NkAGO1* and *NkAGO10* in the dissected tissues of the different stages of *N. khasiana* leaf development. The $2^{-\Delta Ct}$ values were generated for each gene and plotted against the dissected tissues.

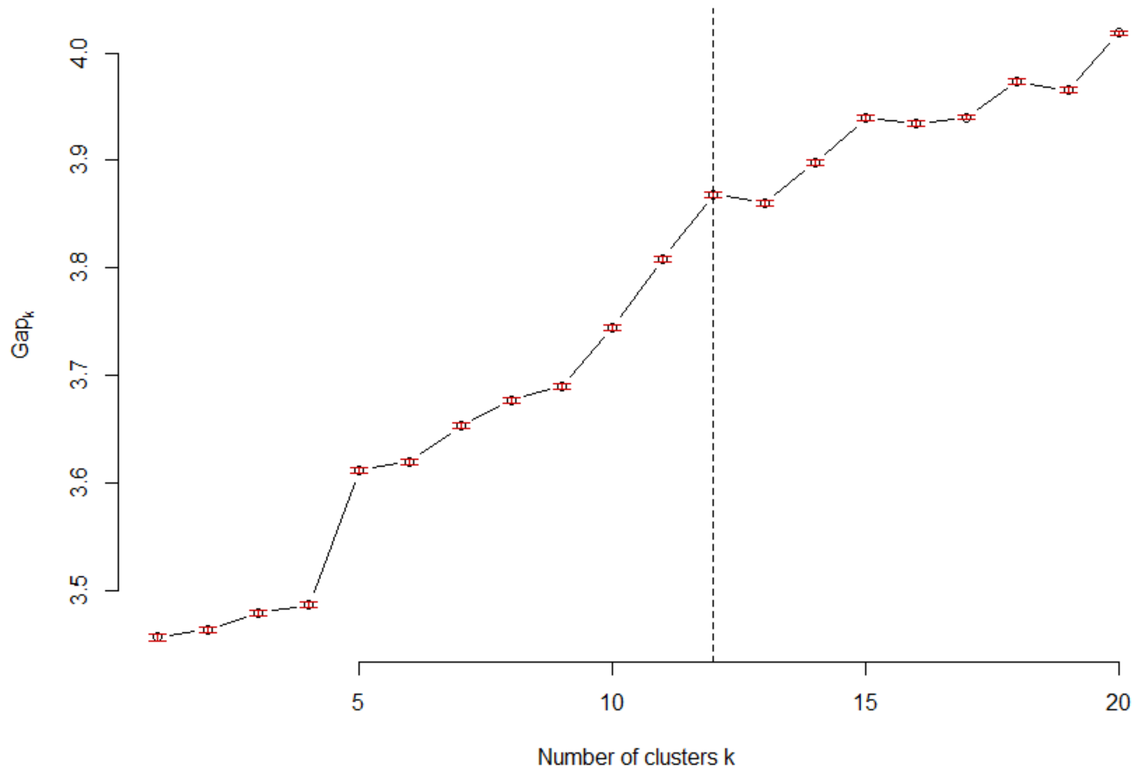

Fig. S7. Determination of the number of clusters for k-means clustering. The number of clusters as determined by gap statistic using R is 12.

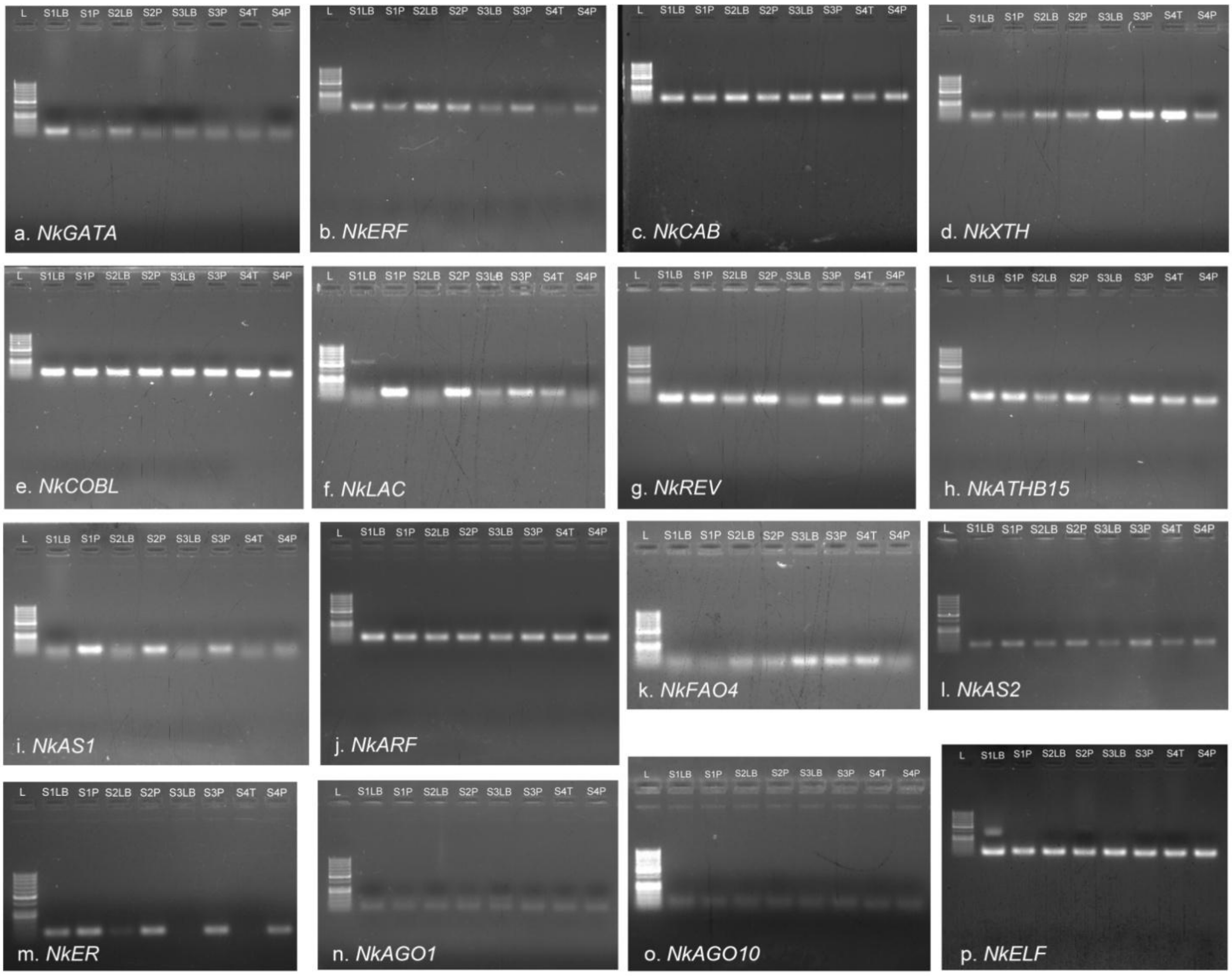

Fig. S8. **a-p**, Full-length gels photos of the selected genes amplified using RT-PCR. RT-PCR analysis was conducted to determine the local expression of selected enriched and/or related genes in the first four developmental stages viz. leaf base (S1LB) and pitcher (S1P) of stage 1, leaf base (S2LB) and pitcher (S2P) of stage 2, leaf base (S3LB) and pitcher (S3P) of stage 3 as well as the tendril (S4T) and pitcher (S4P) of stage 4. Selected genes corresponding to each gel photos are written on the left side at the bottom. Portions of these gels showing just the amplified products were cropped and displayed in Figure 6b. L represents 50bp ladder.

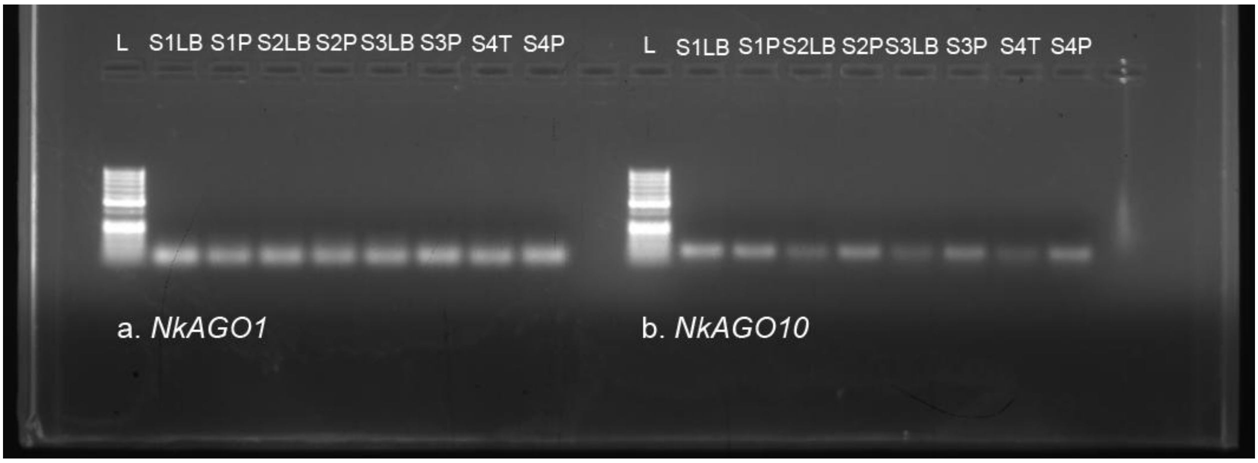

Fig. S9. **a-b,** Full-length gels photos of the selected genes (*NkAGO1* and *NkAGO10*) amplified using RT-PCR at increased cycles of amplification (35 cycles). Portions of these gels showing just the amplified products were cropped and displayed in Fig. S5, Supplementary information. S1LB and S1P - leaf base and pitcher of stage 1, respectively; S2LB and S2P - leaf base and pitcher of stage 2, respectively; S3LB and S3P - leaf base and pitcher of stage 3, respectively; S4T and S4P - tendril and pitcher of stage 4, respectively. L represents 50bp ladder.

Table S1: RNA sequencing statistics of the different stages of *N. khasiana* leaf development. Two biological replicates were sequenced.

Sample	Developmental Stage	Number of raw reads	Number of cleaned paired-end reads	Number of bases (Gb)	Average read length (bases)	% Q >30	Number of reads aligned (paired-end)	% of reads aligned
AS1	Stage 1	31,005,009	23,779,624	3.77	79.28	94.81	20,317,310	85.44
AS2	Stage 2	49,189,329	26,089,633	2.12	81.53	94.63	25,090,400	96.17
AS3	Stage 3	52,197,631	20,555,551	3.34	81.3	94.34	19,850,495	96.57
AS4	Stage 4	55,376,866	46,869,292	7.37	78.68	93.59	44,638,313	95.24
AS5	Stage 5	55,374,606	34,259,133	5.51	80.53	92.35	33,056,637	96.49
CS1	Stage 1	43,991,397	31,0,85,183	4.9	78.92	93.15	27,830,564	89.53
CS2	Stage 2	55,824,459	27,682,488	4.3	77.77	91.96	25,678,275	92.76
CS3	Stage 3	49,406,910	11,932,059	1.81	76.13	90.25	11,380,797	95.38
CS4	Stage 4	54,903,310	35,727,081	5.57	77.96	92.12	33,690,637	94.3
CS5	Stage 5	54,987,699	12,160,072	1.85	76.44	90.3	11,716,229	96.35
		502,257,216	270,140,116			92.75	253,249,657	93.823

Table S2. List of transcripts expressed exclusively in each stage of *N. khasiana* leaf development.

Transcript ID	Transcript length	NCBI Accession	% Identity	E-value	Uniprot Accession	Gene Description
Stage 1						
c169541_g1_i3	433	731360552	85	5.00E-75	A0A0J8BJP6	PREDICTED: endoplasmin homolog [Beta vulgaris subsp. vulgaris]
c169853_g2_i1	238	731349093	95	2.00E-37	A0A0J8BZC2	PREDICTED: histone H2A [Beta vulgaris subsp. vulgaris]
c169853_g2_i2	376	731349093	94	7.00E-57	A0A0J8BZC2	PREDICTED: histone H2A [Beta vulgaris subsp. vulgaris]
c17184_g1_i1	262	731336305	64	6.00E-19	A0A0J8C9N4	PREDICTED: histone H1-like [Beta vulgaris subsp. vulgaris]
c172819_g1_i1	805	731331655	66	3.00E-46	A0A0J8CEW3	PREDICTED: early light-induced protein 2, chloroplastic-like [Beta vulgaris subsp. vulgaris]
c172819_g1_i2	769	731331655	66	2.00E-46	A0A0J8CEW3	PREDICTED: early light-induced protein 2, chloroplastic-like [Beta vulgaris subsp. vulgaris]
c17284_g1_i1	440	731338098	78	5.00E-69	A0A0J8CA65	PREDICTED: oxygen-evolving enhancer protein 2, chloroplastic [Beta vulgaris subsp. vulgaris]
c174033_g1_i1	815	902207442	56	3.00E-72	A0A0K9R898	hypothetical protein SOVF_095730 [Spinacia oleracea]
c178339_g3_i1	261	870858605	62	1.00E-07	A0A0J8CDN8	hypothetical protein BVRB_5g118560 [Beta vulgaris subsp. vulgaris]
c18036_g1_i1	366	731328152	88	2.00E-71	A0A0J8FC99	PREDICTED: cysteine synthase [Beta vulgaris subsp. vulgaris]
c181688_g12_i1	209	902207462	66	4.00E-19	A0A0K9R896	hypothetical protein SOVF_095690, partial [Spinacia oleracea]
c184807_g4_i1	204	68685649	45	2.00E-17	Q3I6J4	pol-polyprotein [Silene latifolia]
c185673_g1_i1	286	902223048	73	3.00E-17	A0A0K9RIM4	hypothetical protein SOVF_062300 [Spinacia oleracea]
c185986_g1_i1	366	902234308	74	4.00E-35	A0A0K9RWV4	hypothetical protein SOVF_025150 [Spinacia oleracea]
c186258_g1_i1	440	870756954	60	9.00E-24	A0A0J7YMN1	hypothetical protein BVRB_041940 [Beta vulgaris subsp. vulgaris]
c187415_g1_i1	208	731357957	84	8.00E-10	A0A0J8BNF7	PREDICTED: serine/arginine-rich splicing factor SC35 [Beta vulgaris subsp. vulgaris]
c187465_g1_i1	217	731375740	84	8.00E-35	A0A0J8B2U8	PREDICTED: L-ascorbate oxidase homolog [Beta vulgaris subsp. vulgaris]
c188184_g1_i1	318	902238787	76	3.00E-15	A0A0K9S2G1	hypothetical protein SOVF_007620, partial [Spinacia oleracea]
c188805_g1_i1	398	731359185	69	3.00E-43	A0A0J8BI68	PREDICTED: phospholipase D alpha 1-like [Beta vulgaris subsp. vulgaris]
c1889_g1_i1	617	870866938	45	2.00E-08	A0A0J8CVU8	hypothetical protein BVRB_2g033830 [Beta vulgaris subsp. vulgaris]
c189574_g1_i1	444	731362405	58	1.00E-37	A0A0J8BHK5	PREDICTED: basic blue protein-like [Beta vulgaris subsp. vulgaris]
c191554_g1_i1	252	902217641	66	1.00E-08	A0A0K9RG66	hypothetical protein SOVF_076460 [Spinacia oleracea]
c191586_g1_i1	228	731377096	82	3.00E-35	A0A0J8B5Q5	PREDICTED: glycine dehydrogenase (decarboxylating), mitochondrial [Beta vulgaris subsp. vulgaris]
c193148_g1_i1	211	902208228	84	2.00E-34	A0A0K9RA51	hypothetical protein SOVF_094190 [Spinacia oleracea]
c194797_g1_i1	215	731366257	64	7.00E-12	A0A0J8BDJ4	PREDICTED: UTP--glucose-1-phosphate uridylyltransferase [Beta vulgaris subsp. vulgaris]
c196602_g1_i1	403	731332702	82	8.00E-71	A0A0J8F5W1	PREDICTED: syntaxin-22 isoform X1 [Beta vulgaris subsp. vulgaris]
c198715_g1_i1	228	902150139	71	2.00E-30	A0A0K9QDN7	hypothetical protein SOVF_190930 [Spinacia oleracea]
c198891_g1_i1	437	870861857	46	4.00E-23	A0A0J8FB77	hypothetical protein BVRB_4g086580 [Beta vulgaris subsp. vulgaris]
c200373_g1_i1	251	731336807	95	7.00E-52	A0A0J8C8M6	PREDICTED: ADP-ribosylation factor 2-B-like [Beta vulgaris subsp. vulgaris]
c200547_g1_i1	351	731330577	75	8.00E-36	A0A0J8CGB8	PREDICTED: FAM10 family protein At4g22670 [Beta vulgaris subsp. vulgaris]
c201769_g1_i1	275	731357520	58	4.00E-29	A0A0J8BMK2	PREDICTED: probable flavin-containing monooxygenase 1 [Beta vulgaris subsp. vulgaris]
c205074_g1_i1	251	731316843	51	1.00E-19	A0A0J8D4G2	PREDICTED: chaperone protein ClpB4, mitochondrial [Beta vulgaris subsp. vulgaris]
c205204_g1_i1	255	731325579	100	5.00E-45	A0A0J8CL16	PREDICTED: histone H3.2 [Beta vulgaris subsp. vulgaris]
c205491_g1_i1	732	902205504	67	7.00E-99	A0A0K9R6P5	hypothetical protein SOVF_100540 [Spinacia oleracea]
c206124_g1_i1	278	731353317	83	7.00E-34	A0A0J8BNL2	PREDICTED: uncharacterized protein LOC104902040 [Beta vulgaris subsp. vulgaris]
c207567_g1_i1	377	731359783	94	6.00E-79	A0A0J8ED79	PREDICTED: heat shock 70 kDa protein, mitochondrial [Beta vulgaris subsp. vulgaris]
c207629_g1_i1	203	19699051	86	3.00E-25	Q8RXA8	nucleoside diphosphate kinase III [Spinacia oleracea]
c208828_g1_i1	241	731348555	95	1.00E-47	A0A0J8BVC6	PREDICTED: probable pyridoxal biosynthesis protein PDX1 [Beta vulgaris subsp. vulgaris]
c209212_g1_i1	213	902233050	82	3.00E-24	A0A0K9RTN6	hypothetical protein SOVF_029960 [Spinacia oleracea]
c209555_g1_i1	241	902170106	81	7.00E-16	A0A0K9QKK7	hypothetical protein SOVF_168790 [Spinacia oleracea]
c209636_g1_i1	370	731335404	83	2.00E-71	A0A0J8CEG7	PREDICTED: cytochrome c1-2, heme protein, mitochondrial [Beta vulgaris subsp. vulgaris]
c209998_g1_i1	224	731333072	55	9.00E-12	A0A0J8CD75	PREDICTED: protein notum homolog isoform X1 [Beta vulgaris subsp. vulgaris]
c210314_g1_i1	235	731364722	63	1.00E-15	A0A0J8BF45	PREDICTED: 50S ribosomal protein L18, chloroplastic [Beta vulgaris subsp. vulgaris]
c210463_g1_i1	283	731332454	69	3.00E-29	A0A0J8CI46	PREDICTED: ATP synthase subunit epsilon, mitochondrial [Beta vulgaris subsp. vulgaris]
c21061_g1_i2	205	731364908	90	3.00E-18	A0A0J8BEW7	PREDICTED: probable histone H2A.5 [Beta vulgaris subsp. vulgaris]

c211020_g1_i1	275	731356662	68	4.00E-28	A0A0J8BPN6	PREDICTED: 26S proteasome non-ATPase regulatory subunit 2 homolog A-like [Beta vulgaris subsp. vulgaris]
c215870_g1_i1	247	902192548	70	6.00E-36	A0A0K9QZU9	hypothetical protein SOVF_129040 isoform A [Spinacia oleracea]
c216953_g1_i1	217	731368143	77	6.00E-20	A0A0J8B8C3	PREDICTED: vignain-like [Beta vulgaris subsp. vulgaris]
c219508_g1_i1	214	731351930	57	2.00E-07	A0A0J8ELB3	PREDICTED: uncharacterized protein DS12 from 2D-PAGE of leaf, chloroplastic [Beta vulgaris subsp. vulgaris]
c219830_g1_i1	209	731361751	90	7.00E-39	A0A0J8BI03	PREDICTED: proteasome subunit alpha type-5-like [Beta vulgaris subsp. vulgaris]
c220693_g1_i1	205	902173404	47	3.00E-05	A0A0K9QLX7	hypothetical protein SOVF_164230, partial [Spinacia oleracea]
c220955_g1_i1	265	902203036	70	2.00E-25	A0A0K9R4N4	hypothetical protein SOVF_107430 [Spinacia oleracea]
c221007_g1_i1	221	731317214	78	2.00E-31	A0A0J8CWD8	PREDICTED: ER membrane protein complex subunit 1 [Beta vulgaris subsp. vulgaris]
c221964_g1_i1	220	731317158	88	3.00E-16	A0A0J8FQV5	PREDICTED: coiled-coil domain-containing protein 25 [Beta vulgaris subsp. vulgaris]
c225113_g1_i1	404	902194915	45	5.00E-28	A0A0K9QZM1	hypothetical protein SOVF_123530 [Spinacia oleracea]
c225413_g1_i1	205	902226416	72	6.00E-29	A0A0K9RNE1	hypothetical protein SOVF_053180 [Spinacia oleracea]
c225539_g1_i1	360	870869355	56	2.00E-16	A0A0J8D1T1	hypothetical protein BVRB_1g001190 [Beta vulgaris subsp. vulgaris]
c225621_g1_i1	229	902190789	48	1.00E-11	A0A0K9QWP6	hypothetical protein SOVF_132800, partial [Spinacia oleracea]
c225836_g1_i1	242	870748244	46	4.00E-15	A0A0J8BFE5	hypothetical protein BVRB_019140 [Beta vulgaris subsp. vulgaris]
c225921_g1_i1	207	902234435	53	1.00E-10	A0A0K9RV92	hypothetical protein SOVF_024700 [Spinacia oleracea]
c225950_g1_i1	252	731332832	85	7.00E-45	A0A0J8CH28	PREDICTED: phosphoribulokinase, chloroplastic [Beta vulgaris subsp. vulgaris]
c226169_g1_i1	270	359372825	56	2.00E-16	G9FIF2	hypothetical protein [Beta vulgaris]
c226970_g1_i1	232	902177082	83	8.00E-44	A0A0K9QNS4	hypothetical protein SOVF_158530 [Spinacia oleracea]
c227073_g1_i1	224	902240628	82	7.00E-34	A0A0K9S2Q8	hypothetical protein SOVF_001040 isoform B [Spinacia oleracea]
c227181_g1_i1	408	902163900	80	2.00E-71	A0A0K9QI05	hypothetical protein SOVF_176840 isoform A [Spinacia oleracea]
c227477_g1_i1	291	731331438	48	2.00E-16	A0A0J8CJH2	PREDICTED: uncharacterized protein LOC104892429 isoform X1 [Beta vulgaris subsp. vulgaris]
c228207_g1_i1	209	731339820	42	9.00E-07	A0A0J8C4C3	PREDICTED: probable ATP synthase 24 kDa subunit, mitochondrial [Beta vulgaris subsp. vulgaris]
c229163_g1_i1	218	731378359	76	1.00E-30	A0A0J8DVI3	PREDICTED: uncharacterized protein LOC104885143 [Beta vulgaris subsp. vulgaris]
c230476_g1_i1	204	731351875	46	8.00E-13	A0A0J8BQG7	PREDICTED: cell division cycle protein 48 homolog [Beta vulgaris subsp. vulgaris]
c231900_g1_i1	212	731325608	57	9.00E-14	A0A0J8FF42	PREDICTED: zinc finger A20 and AN1 domain-containing stress-associated protein 8 [Beta vulgaris subsp. vulgaris]
c232308_g1_i1	281	731327692	79	3.00E-30	A0A0J8CJE5	PREDICTED: cytochrome c oxidase subunit 5C [Beta vulgaris subsp. vulgaris]
c232426_g1_i1	322	219741568	86	6.00E-63	A0A0K9RIA8	unnamed protein product [Spinacia oleracea]
c232617_g1_i1	218	731377096	92	6.00E-40	A0A0J8B5Q5	PREDICTED: glycine dehydrogenase (decarboxylating), mitochondrial [Beta vulgaris subsp. vulgaris]
c23265_g1_i1	333	870840040	53	2.00E-09	A0A0J8DV88	hypothetical protein BVRB_015590 [Beta vulgaris subsp. vulgaris]
c232822_g1_i1	201	902218337	52	2.00E-11	A0A0K9REU3	hypothetical protein SOVF_074710 [Spinacia oleracea]
c232837_g1_i1	228	902184738	86	5.00E-32	A0A0K9QSW1	hypothetical protein SOVF_145120 [Spinacia oleracea]
c233185_g1_i1	268	731324218	70	5.00E-24	A0A0J8CSL7	PREDICTED: villin-3 isoform X1 [Beta vulgaris subsp. vulgaris]
c234048_g1_i1	325	870861857	45	1.00E-24	A0A0J8FB77	hypothetical protein BVRB_4g086580 [Beta vulgaris subsp. vulgaris]
c23435_g1_i1	488	731318998	43	2.00E-20	A0A0J8CUG2	PREDICTED: mediator of RNA polymerase II transcription subunit 15a [Beta vulgaris subsp. vulgaris]
c23435_g1_i2	377	902190536	74	3.00E-16	A0A0K9QWM1	hypothetical protein SOVF_132680 [Spinacia oleracea]
c23509_g1_i1	396	197693506	55	5.00E-08	B5M104	cytochrome c oxidase subunit 3, partial (mitochondrion) [Opuntia matudae]
c235468_g1_i1	273	755802	67	5.00E-11	A0A0K9QRK4	16 kDa protein of the photosynthetic oxygen-evolving protein (OEC) [Spinacia oleracea]
c235531_g1_i1	260	902183362	43	6.00E-08	A0A0K9QTU5	hypothetical protein SOVF_147540, partial [Spinacia oleracea]
c236238_g1_i1	504	902161257	56	8.00E-56	A0A0K9QIY8	hypothetical protein SOVF_180000 [Spinacia oleracea]
c236327_g1_i1	240	731313914	49	7.00E-18	A0A0J8D5E2	PREDICTED: GDSL esterase/lipase At1g33811 [Beta vulgaris subsp. vulgaris]
c23646_g1_i1	225	2369714	91	9.00E-43	O23755	elongation factor 2 [Beta vulgaris subsp. vulgaris]
c237053_g1_i1	768	902196700	48	2.00E-28	A0A0K9R0Y9	hypothetical protein SOVF_119120 [Spinacia oleracea]
c237266_g1_i1	311	902232594	91	8.00E-30	A0A0K9RUW5	hypothetical protein SOVF_031810 [Spinacia oleracea]
c237335_g1_i1	260	902211052	87	6.00E-05	A0A0K9RTR6	hypothetical protein SOVF_087770 [Spinacia oleracea]
c237713_g1_i1	322	902218055	46	6.00E-08	A0A0K9RG48	hypothetical protein SOVF_075250 [Spinacia oleracea]
c239268_g1_i1	201	731375185	69	5.00E-12	A0A0J8B704	PREDICTED: uncharacterized protein LOC104884251 [Beta vulgaris subsp. vulgaris]
c241233_g1_i1	269	902132832	55	3.00E-14	A0A0K9QAP4	hypothetical protein SOVF_200590 [Spinacia oleracea]
c243196_g1_i1	227	902187074	80	8.00E-37	A0A0K9QUB3	hypothetical protein SOVF_140260 [Spinacia oleracea]

c243540_g1_i1	298	870856272	74	2.00E-23	A0A0J8C2F1	hypothetical protein BVRB_6g145050 [Beta vulgaris subsp. vulgaris]
c244473_g1_i1	214	902175838	87	2.00E-30	A0A0K9QN50	hypothetical protein SOVF_160510 [Spinacia oleracea]
c245497_g1_i1	235	731312905	92	2.00E-43	A0A0J8D298	PREDICTED: uncharacterized protein LOC104892702 [Beta vulgaris subsp. vulgaris]
c246039_g1_i1	269	343172062	80	3.00E-40	G5DW38	alanine-2-oxoglutarate aminotransferase, partial [Silene latifolia]
c24625_g1_i1	353	731325551	58	4.00E-06	A0A0J8CQY6	PREDICTED: linoleate 13S-lipoxygenase 2-1, chloroplastic-like [Beta vulgaris subsp. vulgaris]
c246564_g1_i1	207	902155691	77	2.00E-18	A0A0K9QFB0	hypothetical protein SOVF_185780 [Spinacia oleracea]
c247311_g1_i1	365	731311481	62	7.00E-51	A0A0J8D828	PREDICTED: reticulon-like protein B4 [Beta vulgaris subsp. vulgaris]
c247429_g1_i1	323	731368458	77	5.00E-50	A0A0J8BAY0	PREDICTED: 3-ketoacyl-CoA thiolase 2, peroxisomal [Beta vulgaris subsp. vulgaris]
c247757_g1_i1	242	731328366	89	1.00E-44	A0A0J8CJ46	PREDICTED: UPF0603 protein At1g54780, chloroplastic [Beta vulgaris subsp. vulgaris]
c248177_g1_i1	246	731375349	56	8.00E-19	A0A0J8B6X8	PREDICTED: uncharacterized oxidoreductase At4g09670 [Beta vulgaris subsp. vulgaris]
c249776_g1_i1	304	902229559	81	2.00E-24	A0A0K9RPZ0	hypothetical protein SOVF_042250 [Spinacia oleracea]
c250454_g1_i1	341	902222245	91	8.00E-68	A0A0K9RI15	hypothetical protein SOVF_064530 isoform B [Spinacia oleracea]
c250538_g1_i1	222	902203417	69	8.00E-35	A0A0K9R4V9	hypothetical protein SOVF_106360 [Spinacia oleracea]
c251063_g1_i1	238	870864676	84	1.00E-36	A0A0J8FIR9	hypothetical protein BVRB_3g057460 isoform B [Beta vulgaris subsp. vulgaris]
c252097_g1_i1	212	731361149	56	9.00E-20	A0A0J8BJ01	PREDICTED: eukaryotic translation initiation factor 3 subunit M-like [Beta vulgaris subsp. vulgaris]
c252991_g1_i1	380	731321334	87	3.00E-37	A0A0J8FKL0	PREDICTED: 60S ribosomal protein L9-like [Beta vulgaris subsp. vulgaris]
c255230_g1_i1	296	902217422	49	6.00E-29	A0A0K9RFU6	hypothetical protein SOVF_077600 [Spinacia oleracea]
c255881_g1_i1	357	902147851	71	5.00E-49	A0A0K9QEV2	hypothetical protein SOVF_192630 [Spinacia oleracea]
c256490_g1_i1	203	731360369	61	2.00E-24	A0A0J8BKQ9	PREDICTED: uncharacterized protein LOC104905030 [Beta vulgaris subsp. vulgaris]
c257228_g1_i1	277	731375672	66	5.00E-38	A0A0J8B2V7	PREDICTED: ectonucleotide pyrophosphatase/phosphodiesterase family member 3-like [Beta vulgaris subsp. vulgaris]
c258148_g1_i1	230	731311410	91	1.00E-45	A0A0J8D7K6	PREDICTED: ubiquitin-conjugating enzyme E2 28 [Beta vulgaris subsp. vulgaris]
c258815_g1_i1	236	731352442	55	2.00E-13	A0A0J8BPN4	PREDICTED: uncharacterized protein LOC104901657 [Beta vulgaris subsp. vulgaris]
c260869_g1_i1	203	731339302	41	8.00E-08	A0A0J8C9W8	PREDICTED: cytochrome c oxidase subunit 6a, mitochondrial [Beta vulgaris subsp. vulgaris]
c260937_g1_i1	328	731359634	82	4.00E-48	A0A0J8BIQ1	PREDICTED: costars family protein [Beta vulgaris subsp. vulgaris]
c262569_g1_i1	220	902229419	71	2.00E-29	A0A0K9RPU8	hypothetical protein SOVF_042400 [Spinacia oleracea]
c262633_g1_i1	210	902213507	94	1.00E-41	A0A0K9RE73	hypothetical protein SOVF_081440 [Spinacia oleracea]
c262998_g1_i1	277	731348276	78	2.00E-31	A0A0J8EPK7	PREDICTED: bifunctional nitrilase/nitrile hydratase NIT4A [Beta vulgaris subsp. vulgaris]
c263446_g1_i1	223	115605364	49	4.00E-10	Q002B9	putative thionin precursor [Knorringia sibirica]
c264657_g1_i1	252	902188496	99	3.00E-46	A0A0K9QV69	hypothetical protein SOVF_137210 [Spinacia oleracea]
c265553_g1_i1	237	902176463	90	5.00E-36	A0A0K9QPZ3	hypothetical protein SOVF_159780 [Spinacia oleracea]
c266221_g1_i1	239	731331302	42	2.00E-13	A0A0J8CEF6	PREDICTED: cytochrome P450 86B1-like [Beta vulgaris subsp. vulgaris]
c266749_g1_i1	259	902229233	69	4.00E-36	A0A0K9RPM6	hypothetical protein SOVF_043330 [Spinacia oleracea]
c267422_g1_i1	247	902238836	51	8.00E-06	A0A0K9S2J1	hypothetical protein SOVF_007410 [Spinacia oleracea]
c268738_g1_i1	538	870859108	65	2.00E-82	A0A0J8CAE5	hypothetical protein BVRB_5g116940 [Beta vulgaris subsp. vulgaris]
c268882_g1_i1	271	902237281	53	9.00E-09	A0A0K9S0V2	hypothetical protein SOVF_013130 isoform B [Spinacia oleracea]
c269408_g1_i1	265	902156530	90	1.00E-43	A0A0K9QH63	hypothetical protein SOVF_184920 [Spinacia oleracea]
c270399_g1_i1	235	902217949	79	1.00E-37	A0A0K9RG17	hypothetical protein SOVF_075440 [Spinacia oleracea]
c271493_g1_i1	225	731320011	85	9.00E-40	A0A0J8CY46	PREDICTED: proteasome subunit beta type-3-A [Beta vulgaris subsp. vulgaris]
c272668_g1_i1	329	731310585	76	7.00E-05	A0A0J8D1H4	PREDICTED: selenoprotein K [Beta vulgaris subsp. vulgaris]
c272924_g1_i1	307	731326692	47	3.00E-24	A0A0J8CP46	PREDICTED: dnaJ homolog subfamily B member 3 [Beta vulgaris subsp. vulgaris]
c273064_g1_i1	222	902236963	66	2.00E-27	A0A0K9RYP0	hypothetical protein SOVF_014190 [Spinacia oleracea]
c273216_g1_i1	294	902191303	87	6.00E-43	A0A0K9QZ58	hypothetical protein SOVF_131150 [Spinacia oleracea]
c273382_g1_i1	261	731352483	65	2.00E-33	A0A0J8EKA4	PREDICTED: uncharacterized protein LOC104901676 [Beta vulgaris subsp. vulgaris]
c275519_g1_i1	205	731365470	91	8.00E-37	A0A0J8BBL3	PREDICTED: multiprotein-bridging factor 1b [Beta vulgaris subsp. vulgaris]
c275617_g1_i1	225	731321454	77	2.00E-26	A0A0J8CQS5	PREDICTED: cytochrome b5 isoform E [Beta vulgaris subsp. vulgaris]
c276077_g1_i1	290	1488647	71	5.00E-40	Q41382	RNA helicase [Spinacia oleracea]
c277033_g1_i1	253	870858605	51	4.00E-18	A0A0J8CDN8	hypothetical protein BVRB_5g118560 [Beta vulgaris subsp. vulgaris]
c278823_g1_i1	222	870830417	62	4.00E-05	A0A0J8AZK0	hypothetical protein BVRB_023710 [Beta vulgaris subsp. vulgaris]
c281255_g1_i1	288	902195546	68	6.00E-25	A0A0K9R013	hypothetical protein SOVF_122190 [Spinacia oleracea]
c281637_g1_i1	272	902220217	67	1.00E-27	A0A0K9RGB2	hypothetical protein SOVF_069950 [Spinacia oleracea]

c282332_g1_i1	288	902229444	74	5.00E-33	A0A0K9RPV3	hypothetical protein SOVF_042500 [Spinacia oleracea]
c282918_g1_i1	236	731337148	63	2.00E-20	A0A0J8C803	PREDICTED: mitochondrial outer membrane protein porin 2-like [Beta vulgaris subsp. vulgaris]
c283826_g1_i1	222	902155157	90	4.00E-17	A0A0K9QF21	hypothetical protein SOVF_186280 [Spinacia oleracea]
c284819_g1_i1	311	731345443	79	3.00E-51	A0A0J8C250	PREDICTED: pyruvate kinase, cytosolic isozyme-like [Beta vulgaris subsp. vulgaris]
c286702_g1_i1	294	902219589	83	3.00E-45	A0A0K9RHD5	hypothetical protein SOVF_071570 [Spinacia oleracea]
c288040_g1_i1	256	731335400	85	7.00E-40	A0A0J8CEX0	PREDICTED: nucleobase-ascorbate transporter 6 [Beta vulgaris subsp. vulgaris]
c288188_g1_i1	365	902230318	69	1.00E-29	A0A0K9RQJ0	hypothetical protein SOVF_039790 [Spinacia oleracea]
c288504_g1_i1	235	731342891	86	3.00E-40	A0A0J8C0M8	PREDICTED: nucleobase-ascorbate transporter 7-like [Beta vulgaris subsp. vulgaris]
c288544_g1_i1	266	731365016	46	2.00E-11	A0A0J8E5T6	PREDICTED: uncharacterized protein LOC104907109 [Beta vulgaris subsp. vulgaris]
c292201_g1_i1	284	731330577	63	2.00E-14	A0A0J8CGB8	PREDICTED: FAM10 family protein At4g22670 [Beta vulgaris subsp. vulgaris]
c292738_g1_i1	219	731327076	42	5.00E-08	A0A0J8CKC3	PREDICTED: aspartic proteinase nepenthesin-1 [Beta vulgaris subsp. vulgaris]
c294180_g1_i1	299	731313641	72	3.00E-45	A0A0J8D0F9	PREDICTED: peroxidase 12-like [Beta vulgaris subsp. vulgaris]
c295372_g1_i1	391	731341153	91	3.00E-65	A0A0J8C2U7	PREDICTED: stromal 70 kDa heat shock-related protein, chloroplastic [Beta vulgaris subsp. vulgaris]
c295663_g1_i1	218	92090520	81	6.00E-35	Q1PCS4	SUS1, partial [Dianthus caryophyllus]
c295698_g1_i1	239	902094619	85	2.00E-41	A0A0K9QA11	hypothetical protein SOVF_209850 [Spinacia oleracea]
c296439_g1_i1	315	902207265	71	3.00E-49	A0A0K9R847	hypothetical protein SOVF_096160, partial [Spinacia oleracea]
c29694_g1_i1	216	870854621	71	2.00E-25	A0A0J8ET61	hypothetical protein BVRB_7g160560 [Beta vulgaris subsp. vulgaris]
c297483_g1_i1	216	731323488	89	2.00E-36	A0A0J8CU14	PREDICTED: 2-isopropylmalate synthase A-like isoform X2 [Beta vulgaris subsp. vulgaris]
c298035_g1_i1	311	902223660	73	8.00E-48	A0A0K9RJ52	hypothetical protein SOVF_060820 [Spinacia oleracea]
c298928_g1_i1	245	731363761	81	3.00E-39	A0A0J8BG62	PREDICTED: peroxisomal fatty acid beta-oxidation multifunctional protein MFP2 [Beta vulgaris subsp. vulgaris]
c300103_g1_i1	246	731316767	83	3.00E-46	A0A0J8D3W8	PREDICTED: 40S ribosomal protein S11 [Beta vulgaris subsp. vulgaris]
c300891_g1_i1	201	731372992	45	9.00E-09	A0A0J8B523	PREDICTED: probable starch synthase 4, chloroplastic/amyloplastic [Beta vulgaris subsp. vulgaris]
c301417_g1_i1	319	902191287	67	2.00E-12	A0A0K9QZ54	hypothetical protein SOVF_131670 [Spinacia oleracea]
c301839_g1_i1	408	902101913	59	2.00E-42	A0A0K9QA16	hypothetical protein SOVF_208120 [Spinacia oleracea]
c302087_g1_i1	292	343172669	98	2.00E-62	G5DWZ1	succinate dehydrogenase [ubiquinone] flavoprotein subunit 1, partial [Silene latifolia]
c302678_g1_i1	294	870857976	53	7.00E-32	A0A0J8C7I1	hypothetical protein BVRB_6g129700 [Beta vulgaris subsp. vulgaris]
c303008_g1_i1	317	731365988	64	3.00E-45	A0A0J8E4L2	PREDICTED: probable glutathione S-transferase [Beta vulgaris subsp. vulgaris]
c303131_g1_i1	310	343172706	73	1.00E-05	G5DX09	calcium-binding EF-hand domain-containing protein, partial [Silene latifolia]
c303519_g1_i1	436	731322888	67	5.00E-58	A0A0J8FII5	PREDICTED: uncharacterized protein LOC104888769 [Beta vulgaris subsp. vulgaris]
c303526_g1_i1	293	731321402	57	1.00E-19	A0A0J8CS99	PREDICTED: caffeic acid 3-O-methyltransferase [Beta vulgaris subsp. vulgaris]
c30433_g1_i1	210	870861302	62	6.00E-09	A0A0J8CGD8	hypothetical protein BVRB_5g098360 isoform B [Beta vulgaris subsp. vulgaris]
c305435_g1_i1	260	902202091	70	4.00E-33	A0A0K9R3T3	hypothetical protein SOVF_110040 [Spinacia oleracea]
c305963_g1_i1	333	92090520	87	6.00E-51	Q1PCS4	SUS1, partial [Dianthus caryophyllus]
c306777_g1_i1	350	902161133	46	6.00E-34	A0A0K9QH25	hypothetical protein SOVF_179960 [Spinacia oleracea]
c307132_g1_i1	218	902154559	49	7.00E-18	A0A0K9QEX4	hypothetical protein SOVF_186800 [Spinacia oleracea]
c307412_g1_i1	280	731334686	51	1.00E-08	A0A0J8CBD9	PREDICTED: ATP-dependent Clp protease ATP-binding subunit clpA homolog CD4B, chloroplastic [Beta vulgaris subsp. vulgaris]
c307481_g1_i1	233	731311531	58	7.00E-20	A0A0J8FXN3	PREDICTED: protein BPS1, chloroplastic-like [Beta vulgaris subsp. vulgaris]
c307559_g1_i1	401	731361394	56	3.00E-43	A0A0J8BGQ9	PREDICTED: xylem serine proteinase 1 [Beta vulgaris subsp. vulgaris]
c30970_g1_i1	201	391225621	53	9.00E-06	I6QMJ0	HMA5-2, partial [Silene vulgaris]
c309880_g1_i1	266	902198775	75	5.00E-21	A0A0K9R2Q6	hypothetical protein SOVF_113490 [Spinacia oleracea]
c309919_g1_i1	226	197312871	74	7.00E-07	B5M1W7	copper homeostasis factor [Rheum australe]
c310035_g1_i1	244	902235112	71	4.00E-05	A0A0K9RWE4	hypothetical protein SOVF_022370 [Spinacia oleracea]
c310281_g1_i1	219	902214714	85	1.00E-10	A0A0K9RDF0	hypothetical protein SOVF_079680 [Spinacia oleracea]
c311834_g1_i1	213	731338518	68	9.00E-25	A0A0J8CBC0	PREDICTED: probable mannitol dehydrogenase [Beta vulgaris subsp. vulgaris]
c312200_g1_i1	206	808692414	84	4.00E-32	A0A0F6QEV7	SDM1 [Silene dioica]
c313450_g1_i1	245	121592411	78	2.00E-24	A2I993	cold-induced phosphoenolpyruvate carboxykinase-like protein [Cerastium arcticum]
c313583_g1_i1	304	731312901	51	7.00E-30	A0A0J8D177	PREDICTED: major pollen allergen Lol p 11-like [Beta vulgaris subsp. vulgaris]
c314353_g1_i1	228	902149709	81	1.00E-37	A0A0K9QF90	hypothetical protein SOVF_191300, partial [Spinacia oleracea]
c315155_g1_i1	279	902223591	82	1.00E-20	A0A0K9RJ34	hypothetical protein SOVF_060390 [Spinacia oleracea]

c315974_g1_i1	429	731357299	67	8.00E-65	A0A0J8BP66	PREDICTED: GDSL esterase/lipase APG-like [Beta vulgaris subsp. vulgaris]
c316947_g1_i1	315	391225621	45	9.00E-07	I6QMJ0	HMA5-2, partial [Silene vulgaris]
c317546_g1_i1	244	870853740	64	9.00E-30	A0A0J8BWR5	hypothetical protein BVRB_7g167480 [Beta vulgaris subsp. vulgaris]
c318193_g1_i1	246	731338711	57	9.00E-24	A0A0J8C6K6	PREDICTED: salicylic acid-binding protein 2-like [Beta vulgaris subsp. vulgaris]
c318850_g1_i1	309	33149681	97	6.00E-14	Q5QJY9	pyruvate decarboxylase [Dianthus caryophyllus]
c319850_g1_i1	243	7007263	90	2.00E-46	Q9LWB7	sucrose synthase [Oxybasis rubra]
c320349_g1_i1	226	359372874	73	7.00E-16	G9FZ36	beta carbonic anhydrase 2 [Mesembryanthemum nodiflorum]
c322524_g1_i1	202	902173928	82	2.00E-31	A0A0K9QM38	hypothetical protein SOVF_163610 [Spinacia oleracea]
c323267_g1_i1	265	902192973	89	4.00E-29	A0A0K9QZP6	hypothetical protein SOVF_128300 [Spinacia oleracea]
c323786_g1_i1	203	731344801	86	2.00E-32	A0A0J8BYR6	PREDICTED: protein transport protein SEC23-like [Beta vulgaris subsp. vulgaris]
c324370_g1_i1	309	902220275	84	9.00E-60	A0A0K9RGC8	hypothetical protein SOVF_069530 [Spinacia oleracea]
c324751_g1_i1	225	870848340	44	2.00E-05	A0A0J8BKN9	hypothetical protein BVRB_9g219220 [Beta vulgaris subsp. vulgaris]
c326196_g1_i1	207	902164838	65	8.00E-30	A0A0K9QIE3	hypothetical protein SOVF_175860 [Spinacia oleracea]
c326397_g1_i1	208	7007263	88	1.00E-31	Q9LWB7	sucrose synthase [Oxybasis rubra]
c326515_g1_i1	212	870857066	51	4.00E-17	A0A0J8C533	hypothetical protein BVRB_6g139190 [Beta vulgaris subsp. vulgaris]
c326981_g1_i1	216	870855114	74	1.00E-31	A0A0J8C023	hypothetical protein BVRB_6g151880 [Beta vulgaris subsp. vulgaris]
c327068_g1_i1	249	440356730	71	2.00E-06	L7R9S0	phosphate transporter, partial [Mesembryanthemum crystallinum]
c327993_g1_i1	221	870849534	87	8.00E-40	A0A0J8BP98	hypothetical protein BVRB_9g210550 [Beta vulgaris subsp. vulgaris]
c329630_g1_i1	251	731332298	78	3.00E-28	A0A0J8CDE4	PREDICTED: bax inhibitor 1-like [Beta vulgaris subsp. vulgaris]
c329781_g1_i1	209	731316567	67	1.00E-06	A0A0J8D422	PREDICTED: DEAD-box ATP-dependent RNA helicase 56 isoform X1 [Beta vulgaris subsp. vulgaris]
c33147_g2_i1	288	7592730	87	1.00E-56	Q9MB82	plasma membrane H+-ATPase, partial [Nepenthes alata]
c34342_g1_i1	285	870840520	72	2.00E-27	A0A0J8B1E7	hypothetical protein BVRB_014810 [Beta vulgaris subsp. vulgaris]
c37023_g1_i1	417	902198017	88	6.00E-47	A0A0K9R237	hypothetical protein SOVF_115380 [Spinacia oleracea]
c3851_g1_i1	336	870749418	90	9.00E-65	A0A0J7YLM1	hypothetical protein BVRB_018790, partial [Beta vulgaris subsp. vulgaris]
c38585_g1_i1	237	13235340	96	5.00E-46	Q9AVU8	putative vacuolar ATP Synthase subunit A [Mesembryanthemum crystallinum]
c40790_g1_i1	310	731356552	83	1.00E-34	A0A0J8BPP6	PREDICTED: shaggy-related protein kinase eta [Beta vulgaris subsp. vulgaris]
c4136_g1_i1	230	870860982	75	9.00E-33	A0A0J8CEQ0	hypothetical protein BVRB_5g102310 [Beta vulgaris subsp. vulgaris]
c44390_g1_i1	357	731355347	84	1.00E-61	A0A0J8EH23	PREDICTED: ruBisCO large subunit-binding protein subunit alpha [Beta vulgaris subsp. vulgaris]
c46359_g1_i1	388	902141745	54	2.00E-28	A0A0K9QBS1	hypothetical protein SOVF_197030 isoform B [Spinacia oleracea]
c4720_g1_i1	241	731360464	94	2.00E-52	A0A0J8EB18	PREDICTED: ubiquitin-fold modifier-conjugating enzyme 1 [Beta vulgaris subsp. vulgaris]
c47244_g1_i1	350	870848131	59	4.00E-39	A0A0J8BG85	hypothetical protein BVRB_9g217240 [Beta vulgaris subsp. vulgaris]
c47424_g1_i1	202	902221260	52	2.00E-12	A0A0K9RH99	hypothetical protein SOVF_066560 [Spinacia oleracea]
c50524_g1_i1	271	731341633	81	1.00E-48	A0A0J8C364	PREDICTED: serine carboxypeptidase-like [Beta vulgaris subsp. vulgaris]
c51287_g1_i1	247	902204604	100	5.00E-45	A0A0K9R803	hypothetical protein SOVF_102870 [Spinacia oleracea]
c52181_g1_i1	404	731350516	70	2.00E-42	A0A0J8BW99	PREDICTED: clathrin light chain 2 [Beta vulgaris subsp. vulgaris]
c52572_g1_i1	411	902173153	84	4.00E-52	A0A0K9QNF5	hypothetical protein SOVF_164510 [Spinacia oleracea]
c53330_g1_i1	385	731356233	79	8.00E-71	A0A0J8BQ68	PREDICTED: beta-galactosidase 8 [Beta vulgaris subsp. vulgaris]
c55767_g1_i1	586	2213869	78	2.00E-44	O04683	ferredoxin I precursor [Mesembryanthemum crystallinum]
c56142_g1_i1	327	731316328	63	2.00E-41	A0A0J8CYH4	PREDICTED: uncharacterized protein LOC104907775 [Beta vulgaris subsp. vulgaris]
c57631_g1_i1	211	902234913	80	7.00E-33	A0A0K9RVV7	hypothetical protein SOVF_023110 [Spinacia oleracea]
c58019_g1_i2	249	870848501	52	3.00E-06	A0A0J8BHB2	hypothetical protein BVRB_9g220700 isoform B [Beta vulgaris subsp. vulgaris]
c58403_g1_i1	304	902227681	62	3.00E-09	A0A0K9RMS7	hypothetical protein SOVF_048860 [Spinacia oleracea]
c61490_g1_i1	286	731335751	97	3.00E-15	A0A0J8CAD8	PREDICTED: elongation factor Tu, chloroplastic [Beta vulgaris subsp. vulgaris]
c62350_g1_i1	323	731327848	45	6.00E-27	A0A0J8CP08	PREDICTED: probable linoleate 9S-lipoxygenase 5 [Beta vulgaris subsp. vulgaris]
c63507_g1_i1	311	731344434	76	2.00E-57	A0A0J8BYZ6	PREDICTED: xyloglucan galactosyltransferase KATAMARI1 homolog [Beta vulgaris subsp. vulgaris]
c64273_g1_i1	221	122890420	71	1.00E-30	A2BCU9	aspartic proteinase [Fagopyrum esculentum]
c64993_g1_i1	345	902212878	62	1.00E-31	A0A0K9RCA5	hypothetical protein SOVF_083090 [Spinacia oleracea]
c678_g1_i1	338	902201948	61	6.00E-45	A0A0K9R3Q2	hypothetical protein SOVF_110410 [Spinacia oleracea]
c678_g1_i2	375	902201948	61	6.00E-52	A0A0K9R3Q2	hypothetical protein SOVF_110410 [Spinacia oleracea]
c68009_g1_i1	204	21234	72	2.00E-22	A0A0K9RXI4	acetohydroxy acid reductoisomerase [Spinacia oleracea]
c68909_g1_i1	209	731353566	54	2.00E-11	A0A0J8BSY2	PREDICTED: palmitoyl-acyl carrier protein thioesterase, chloroplastic [Beta vulgaris subsp. vulgaris]

c68916_g1_i1	201	731328056	88	5.00E-37	A0A0J8CJ79	PREDICTED: zeta-carotene desaturase, chloroplastic/chromoplastic [Beta vulgaris subsp. vulgaris]
c69273_g1_i1	215	902205504	92	2.00E-29	A0A0K9R6P5	hypothetical protein SOVF_100540 [Spinacia oleracea]
c7176_g1_i1	289	731365271	41	6.00E-09	A0A0J8BBS2	PREDICTED: HMG-Y-related protein A-like [Beta vulgaris subsp. vulgaris]
c73894_g1_i1	271	902207096	60	2.00E-17	A0A0K9R9G2	hypothetical protein SOVF_096600 [Spinacia oleracea]
c74196_g1_i1	235	731317968	71	2.00E-11	A0A0J8CVG9	PREDICTED: poly(rC)-binding protein 3 [Beta vulgaris subsp. vulgaris]
c74458_g1_i1	230	9049300	49	2.00E-15	Q9MFD2	orf764 (mitochondrion) [Beta vulgaris subsp. vulgaris]
c74974_g1_i1	209	902196612	47	8.00E-12	A0A0K9R0X2	hypothetical protein SOVF_119410 [Spinacia oleracea]
c76371_g1_i1	286	902191927	60	9.00E-18	A0A0K9QZH1	hypothetical protein SOVF_130420 [Spinacia oleracea]
c79137_g1_i1	273	902184494	86	2.00E-53	A0A0K9QSM9	hypothetical protein SOVF_145550 [Spinacia oleracea]
c80499_g1_i1	221	731355245	68	5.00E-31	A0A0J8EHC2	PREDICTED: succinate dehydrogenase [ubiquinone] iron-sulfur subunit 2, mitochondrial-like [Beta vulgaris subsp. vulgaris]
c80752_g1_i1	281	902184394	72	9.00E-39	A0A0K9QUA6	hypothetical protein SOVF_145810 [Spinacia oleracea]
c8083_g1_i1	268	310753563	41	4.00E-05	E7BQD4	gag-pol polyprotein [Silene latifolia]
c82463_g1_i1	232	731335161	68	6.00E-32	A0A0J8F3R3	PREDICTED: protein NRT1/ PTR FAMILY 2.9-like [Beta vulgaris subsp. vulgaris]
c83466_g2_i1	222	902118927	86	4.00E-36	A0A0K9QBN7	hypothetical protein SOVF_204200 [Spinacia oleracea]
c84653_g1_i1	318	7007263	75	5.00E-51	Q9LWB7	sucrose synthase [Oxybasis rubra]
c84653_g2_i1	333	7007263	95	3.00E-20	Q9LWB7	sucrose synthase [Oxybasis rubra]
c8537_g1_i1	238	870846615	50	5.00E-16	A0A0J8BCS5	hypothetical protein BVRB_2g047520 [Beta vulgaris subsp. vulgaris]
c85704_g1_i1	462	731366157	84	4.00E-93	A0A0J8BAX7	PREDICTED: formate dehydrogenase 1, mitochondrial [Beta vulgaris subsp. vulgaris]
c86162_g1_i1	213	870853869	42	6.00E-05	A0A0J8ERK8	hypothetical protein BVRB_7g167300 [Beta vulgaris subsp. vulgaris]
c8618_g1_i1	220	731365775	44	3.00E-06	A0A0J8BBC3	PREDICTED: copper transport protein ATX1-like [Beta vulgaris subsp. vulgaris]
c89681_g2_i1	250	68685649	48	1.00E-07	Q3I6J4	pol-polyprotein [Silene latifolia]
c92557_g1_i1	330	1657948	79	5.00E-13	O24049	MipC [Mesembryanthemum crystallinum]
c93523_g1_i1	247	197312875	80	2.00E-20	B5M1W9	ribosomal protein L36 [Rheum australe]
c94528_g1_i1	253	257637810	92	3.00E-51	Q1PCS6	unnamed protein product [Dianthus caryophyllus]
c95700_g1_i1	398	902233111	89	1.00E-82	A0A0K9RTQ9	hypothetical protein SOVF_029420 [Spinacia oleracea]
c96818_g1_i1	327	313104128	52	5.00E-25	O04979	RecName: Full=Lon protease homolog 2, peroxisomal
c97821_g1_i1	252	384977930	41	8.00E-05	F4MLC7	hypothetical protein (mitochondrion) [Beta vulgaris subsp. maritima]
c98657_g1_i1	377	902181805	64	9.00E-12	A0A0K9QR70	hypothetical protein SOVF_150260 [Spinacia oleracea]
c98686_g1_i1	289	902235956	70	6.00E-40	A0A0K9RXD2	hypothetical protein SOVF_018600 [Spinacia oleracea]
c98842_g1_i1	223	33333385	83	5.00E-31	Q717T4	nodulin-like intrinsic protein NIP1-1 [Atriplex nummularia]
c99130_g1_i1	365	902234741	50	2.00E-22	A0A0K9RVM1	hypothetical protein SOVF_023800 [Spinacia oleracea]
c99253_g1_i1	624	731326885	78	7E-113	A0A0J8FDI2	PREDICTED: GDSL esterase/lipase At5g33370 [Beta vulgaris subsp. vulgaris]
Stage 2						
c201432_g1_i1	258	731351841	51	4.00E-05	A0A0J8EL70	PREDICTED: putative 3,4-dihydroxy-2-butanone kinase isoform X1 [Beta vulgaris subsp. vulgaris]
c214751_g1_i1	298	371927419	48	2.00E-20	Q38712	Chain A, Structure Of Amaranth 11s Proglobulin Seed Storage Protein From Amaranthus Hypochondriacus L.
c230040_g1_i1	226	510937277	52	4.00E-12	R9UBI1	TPI, partial [Silene latifolia]
c239461_g1_i1	391	731335659	48	3.00E-25	A0A0J8CET0	PREDICTED: oleosin 18.2 kDa-like [Beta vulgaris subsp. vulgaris]
c259394_g1_i1	454	731336513	42	2.00E-27	A0A0J8CCH0	PREDICTED: legumin A-like [Beta vulgaris subsp. vulgaris]
c300845_g1_i1	617	29839279	50	9.00E-60	P83004	RecName: Full=13S globulin basic chain
Stage 3						
c187291_g1_i1	233	902230746	59	2.00E-21	A0A0K9RT21	hypothetical protein SOVF_038440 [Spinacia oleracea]
c191818_g1_i1	625	377823072	47	4.00E-05	H6T8M2	NADH dehydrogenase subunit 4, partial (chloroplast) [Blossfeldia liliputana]
c197114_g1_i1	841	164685436	71	7E-107	B0FAB7	cytochrome oxidase subunit I, partial (mitochondrion) [Tamarix parviflora]
c198870_g1_i1	594	902239099	46	5.00E-43	A0A0K9S145	hypothetical protein SOVF_006100 [Spinacia oleracea]
c198935_g1_i1	745	916446300	55	2.00E-57	A0A0K1ZF84	cytochrome c oxidase subunit 3 (mitochondrion) [Silene vulgaris]
c201861_g1_i1	235	343172677	70	4.00E-34	G5DWZ5	40S ribosomal protein S11-1, partial [Silene latifolia]
c203834_g1_i1	267	731318373	82	1.00E-47	A0A0J8D0R0	PREDICTED: 40S ribosomal protein S23 [Beta vulgaris subsp. vulgaris]
c205557_g1_i1	357	902177082	66	3.00E-46	A0A0K9QNS4	hypothetical protein SOVF_158530 [Spinacia oleracea]
c209398_g1_i1	229	902177082	68	1.00E-32	A0A0K9QNS4	hypothetical protein SOVF_158530 [Spinacia oleracea]

c21211_g1_i1	391	296040791	44	2.00E-29	D7RMX6	NADH dehydrogenase subunit 1
c234790_g1_i1	258	731347955	68	3.00E-35	A0A0J8BXT6	PREDICTED: 40S ribosomal protein SA-like [Beta vulgaris subsp. vulgaris]
c240159_g1_i1	201	38426430	41	8.00E-07	Q6SQ16	maturase, partial (mitochondrion) [Tamarix parviflora]
c245046_g1_i1	403	558554615	44	1.00E-20	V5M3B7	RCR3-like cysteine protease [Mirabilis jalapa]
c251940_g1_i1	225	902126042	64	1.00E-29	A0A0K9QA00	hypothetical protein SOVF_202690 [Spinacia oleracea]
c265063_g1_i1	318	357967226	48	7.00E-08	G8E8V1	ATPase subunit 6 (mitochondrion) [Silene conica]
c271076_g1_i1	262	46850048	64	2.00E-22	Q5QG66	cytochrome oxidase subunit I, partial (mitochondrion) [Dionaea muscipula]
c274284_g1_i1	212	1724104	67	2.00E-22	P93254	methionine adenosyltransferase [Mesembryanthemum crystallinum]
c281354_g1_i1	374	357967255	57	9.00E-48	G8E8W1	cytochrome c oxidase subunit 2 (mitochondrion) [Silene conica]
c281788_g1_i1	457	731314684	42	6.00E-06	A0A0J8CZU7	PREDICTED: LIM domain-containing protein WLIM1 [Beta vulgaris subsp. vulgaris]
c289001_g1_i1	225	902183629	54	1.00E-18	A0A0K9QSE1	hypothetical protein SOVF_147080 [Spinacia oleracea]
c306572_g1_i1	224	731315098	55	3.00E-21	A0A0J8FR40	PREDICTED: 60S acidic ribosomal protein P0 [Beta vulgaris subsp. vulgaris]
c309403_g1_i1	318	302748335	51	7.00E-19	E5DLI0	NADH dehydrogenase subunit 5, partial (mitochondrion) [Mollugo verticillata]
c313152_g1_i1	265	296040763	52	2.00E-12	D7RN08	NADH dehydrogenase subunit 5, partial (mitochondrion) [Silene conica]
c313989_g1_i1	243	870867033	49	3.00E-16	A0A0J8CW52	hypothetical protein BVRB_2g034650 [Beta vulgaris subsp. vulgaris]
c314001_g1_i1	216	902219550	83	2.00E-39	A0A0K9RFS6	hypothetical protein SOVF_071350 [Spinacia oleracea]
c314886_g1_i1	247	902158374	62	8.00E-09	A0A0K9QG16	hypothetical protein SOVF_183150 [Spinacia oleracea]
c329703_g1_i1	227	731329943	64	2.00E-20	A0A0J8CH51	PREDICTED: 40S ribosomal protein S8 [Beta vulgaris subsp. vulgaris]
c43754_g1_i1	279	902223821	71	2.00E-14	A0A0K9RKZ3	hypothetical protein SOVF_059910 [Spinacia oleracea]
Stage 4						
c179797_g36_i1	223	902236781	83	5.00E-05	A0A0K9RYC4	hypothetical protein SOVF_014420 isoform B [Spinacia oleracea]
c194045_g1_i1	224	870749389	54	3.00E-06	A0A0J7YLR3	hypothetical protein BVRB_018810, partial [Beta vulgaris subsp. vulgaris]
c326550_g1_i1	209	40353173	45	3.00E-09	Q70YQ0	pol [Beta nana]
Stage 5						
c204227_g1_i1	261	870807846	42	1.00E-08	A0A0J8AWR0	hypothetical protein BVRB_033130, partial [Beta vulgaris subsp. vulgaris]
c210773_g1_i1	306	870746801	41	5.00E-10	A0A0J7YN26	hypothetical protein BVRB_019620, partial [Beta vulgaris subsp. vulgaris]
c277256_g1_i1	264	870751164	61	1.00E-10	A0A0J7YM11	hypothetical protein BVRB_017900 [Beta vulgaris subsp. vulgaris]
c278475_g1_i1	284	731311963	42	7.00E-05	A0A0J8FY97	PREDICTED: LIM domain-containing protein PLIM2c-like [Beta vulgaris subsp. vulgaris]
c325285_g1_i1	284	731348676	43	3.00E-05	A0A0J8EQ59	PREDICTED: RNA polymerase sigma factor sigB-like isoform X1 [Beta vulgaris subsp. vulgaris]
c53014_g1_i1	391	902192118	55	4.00E-35	A0A0K9RI07	hypothetical protein SOVF_130170 [Spinacia oleracea]
c91507_g1_i1	238	731345002	56	6.00E-08	A0A0J8C3C0	PREDICTED: nucleolar GTP-binding protein 1-like [Beta vulgaris subsp. vulgaris]

Table S3. Real-time qPCR validation of the RNA-seq derived expression patterns of randomly selected DEGs.

Transcript ID	Gene ID*	% identity	E-value	RNA-seq (FPKM)					qPCR ($2^{-\Delta CT}$)					R#
				Stage 1	Stage 2	Stage 3	Stage4	Stage5	Stage 1	Stage 2	Stage 3	Stage4	Stage5	
c62032_g1_i1	LTPG2	51	6.00E-10	4.497	3.709	3.821	3.932	694.592	0.809	0.296	0.743	0.271	105.667	0.904
c81404_g1_i1	PR1	84	5.00E-99	0.377	0.147	0.094	0.201	55.396	0.227	0.082	0.118	0.016	3.126	
c177011_g1_i8	MCM6	80	0	12.857	1.791	1.931	31.758	0.271	0.299	0.117	0.068	0.553	0.088	
c171815_g4_i1	GDSL	72	3.00E-42	0.258	0.042	0.097	0.000	166.518	0.861	0.311	0.542	0.006	3.877	
c177256_g4_i1	UBCE	84	2.00E-105	13.301	3.85	1.817	24.764	0.578	0.280	0.328	0.168	2.965	0.114	
c181015_g16_i5	CYT561	64	2.00E-125	2.353	2.237	9.628	14.765	4.763	0.743	0.332	0.761	1.748	0.510	
c61518_g1_i1	SDL	50	8.00E-21	0.338	0.098	45.041	0.682	3.604	46.197	37.490	101.701	0.212	27.336	
c184352_g2_i4	LAC4	71	4.00E-177	1.698	1.090	8.728	13.725	2.524	2.299	1.548	3.752	26.534	2.370	
c181602_g16_i1	MATK	97	8.00E-141	2359.164	5868.800	8505.213	1398.068	2640.043	112.841	268.556	620.556	4.819	350.602	
c174972_g4_i1	CESA7	91	2.00E-35	5.623	3.788	38.781	65.995	8.901	0.146	0.041	0.084	1.406	0.061	
c178719_g1_i6	BCA1	55	6.00E-28	31.422	200.748	350.938	18.917	62.419	0.003	1.245	15.611	3.598	3.470	
c288228_g1_i1	NEPII	96	0	0.538	0.267	0.052	0.009	134.534	0.402	0.161	0.296	0.011	0.512	
c177383_g2_i1	CYTBP	79	4.00E-83	397.855	1409.053	1033.081	156.068	349.281	50.456	82.457	168.280	9.836	68.994	
c175215_g1_i1	MIPC	86	1.00E-131	5.119	6.787	161.337	91.782	38.616	1.010	0.383	1.240	1.686	1.058	
c184712_g7_i1	CYT3	98	1.00E-155	1044.553	1115.416	1183.956	1016.070	1311.969	18.943	9.286	20.040	0.050	18.198	
c179875_g5_i3	PXD	64	5.00E-138	587.577	327.937	97.857	565.685	80.277	0.146	0.041	0.084	1.406	0.061	
c178566_g13_i1	RPS3	99	0	345.100	337.251	275.820	431.720	356.243	14.093	11.453	0.940	9.580	4.680	
c173233_g1_i2	LTI6A	71	1.00E-08	10.957	13.129	29.074	27.717	71.288	0.077	0.127	0.262	0.162	0.187	
c170929_g1_i1	3DDD	65	0	68.184	37.682	8.204	58.122	4.994	0.339	0.115	0.150	0.004	0.104	
c184890_g15_i2	RTRP	66	5.00E-34	131.682	60.318	21.455	48.749	12.016	3.705	1.563	4.485	0.268	1.267	
c189307_g1_i1	APGC2	63	8.00E-177	165.560	54.381	25.151	160.581	4.341	1.289	3.500	4.414	17.676	8.680	
c182739_g3_i13	FKBP19	93	1.00E-17	2.164	4.932	3.385	2.982	2.321	0.001	0.025	0.057	0.050	0.047	
c173352_g1_i1	RBSCO	89	0	183.108	187.908	64.031	121.716	45.336	1.204	0.670	1.384	0.402	0.673	
c179418_g4_i2	CYCU4	74	5.00E-107	12.241	23.851	3.942	12.982	11.156	1.040	1.255	1.919	1.474	3.042	
c179284_g3_i1	CYTO1	76	2.00E-08	9.496	12.963	11.982	12.852	17.105	0.631	0.251	0.347	0.002	0.297	

*The expanded form of each gene, which can be found in Table S4, has been shortened here for convenience, and some may not represent the true abbreviated form. #Overall Pearson Correlation (R) between the RNA-seq and qPCR data.

Table S4. List of randomly selected significantly DEGs and the real time qPCR primers used for the validation of RNA-seq derived transcript expression patterns.

Sl. No.	Gene ID	Protein description	Forward primer (5' – 3')	Reverse primer (5' – 3')
1	LTPG2	Lipid transfer protein 2	CAGCTTGAGCAGCGTTGATC	GTTTACTAGTTTCCCGTTTAGCCTTAGT
2	PR1	Pathogen-related protein 1	GAGAACCTAGCATGGGCATCA	GCTTCTCGTTGACCCACAAGTT
3	MCM6	DNA replication licensing factor MCM6	GCATTGAGGCTGGTGCACTAA	CCATCTTGTCGAACTCATCAATACA
4	GDSL	GDSL esterase lipase EXL3-like	GCCGAGACTGAAGAAAATGAAGA	AGCTTTTTAGCGTGCGTGAGA
5	UBCE	Ubiquitin-conjugating enzyme E2 20	TTTGGATTGACAGCAGTATGGTTCT	TGCTTGGACATTCTTCAGGATAAA
6	CYT561	Cytochrome b561 and DOMON domain-containing protein	TCCAGCAAATTGAATGACAGTGT	GGTTGCCAGATTTCTGAAGCA
7	SDL	Secoisolariciresinol dehydrogenase-like	AGGCAGAGGATGTGGCAGAA	TTTTGCCCACTGATAATTTTGGA
8	LAC4	Laccase 4	GCTGAAGATGGCGTTCGTAGT	ATCCTTTGGTGGTGGCATAATT
9	MATK	maturaseK	TAACCAGCACTGAAAACCGTCTT	GCGGTCAATAAGGTAGGGATCA
10	CESA7	Cellulose synthase A catalytic subunit 7	AGGATTTGATGATGACCAGGAGAT	GGCTGACTGTCCAAATTTCTTTTC
11	BCA1	Chloroplast beta carbonic anhydrase 1	TGGAGACAGCCTCGTATGAAGA	TCAAGGTCGCCCTTCTCACT
12	NEPII	Aspartic proteinase nepenthesin II	CTCTTCTCCCCACGCTTGATA	AGGTCGATTCGGGCATGAA
13	CYTBP	Cytochrome c biogenesis protein	AAAAGAAGCCGCTATGGTGAAA	TCGAACCGAGATGCTCTAGCA
14	MIPC	MipC	AAAAGGAGCGTGGCTATGAACT	ACGCGGAGGAGCTGATAAAA
15	CYT3	Cytochrome oxidase subunit 3	CGAACTCATCCTTGGCTGCTA	TTGCCTCCAACTCAGAACGTT
16	PXD	Putative peroxidase	ACAACACCGCCCCTCTCA	ACCAGGAGGTTGCCGTAGTAGA
17	RPS3	Ribosomal protein S3	GGGTGTCTTCCAGTGGTCTTCT	GGGAGGACGTTGCAGATGTC
18	LTI6A	Hydrophobic protein LTI6A	CACCTTACAGCAGTAGACGGCATA	GATCGGCGTGTTGCTGACT
19	3DDD	3-dehydroquinate dehydratase/shikimate dehydrogenase	TGCCTCTTATTTTATCTTGTGTGGAA	GGCCAAGCTTGTGCATCTAGA
20	RTRP	Reverse transcriptase-like protein	GAGACAGTGGTACAGGAAGTTCGA	GCGGTCGGAAGTACTCTTATGG
21	APGC2	Aspartic protease in Guard Cell 2	TTGGGCAGGCAATAGGAGAA	GTGGCCCTATGTCACTGTTGTC
22	FKBP19	peptidyl-prolyl cis-trans isomerase FKBP19	CCCTTCTTATTCGGGTGTCTGA	TTGCGAGCCAAGAGAAAGGA
23	RBSCO	ruBisCO large subunit-binding protein subunit alpha	TGTGTGTGACGAAGATAAGGATGTT	GGAACCTTCTGGAACCCTTTTT
24	CYCU4	Cyclin-U4-1	TTCCTCCGCCACTCTCTGA	TTGAGAGTCCCGAAGAAATTCC
25	CYTO1	Cytochrome c oxidase subunit 1	GCCCAAAGACTTGAAGCACATC	TTTGTTTCTTCATGGTCGTAACAGTT
Endogenous genes				
28	UBQ	Ubiquitin	CAGGCAGACAAGAAGCACAGAT	GATTTTGCGTGTTTCGTTTCG
29	ELF	Elongation factor	GGCTTGTCCCCGAAGTTGTT	AGCACCTGGGAGACGTGATT

Table S5. List of genes with their corresponding transcripts IDs, descriptions and the RT-PCR primers used for their amplifications.

Sl. No.	Gene	Transcript ID	Gene description	Forward primer (5' – 3')	Reverse primer (5' – 3')
1	*NkGATA*	c180593_g10_i1	GATA transcription factor 15-like	CCAGGAATGCTAAGGCTCAGA	CACTGGGTTGAACATGATGCA
2	*NkERF*	c291812_g1_i1	Ethylene-responsive transcription factor ERF003	CCTCAAGCCATAATCGGTTCA	TGAGATATTCAGTGCCGCAAAG
3	*NkCAB*	c57897_g1_i1	Chlorophyll a-b binding protein 8	AAACCCGTATCGAAAAAAAGCA	TCTATGGGCTCTCAGATATTTGTAATTG
4	*NkXTH*	c171826_g1_i1	Xyloglucan endotransglucosylase/hydrolase protein 28	GAACGGCAGACAGAACCATTTAT	TTTAAATCCTACACTGTGTCCGTACAC
5	*NkCOBL*	c183187_g12_i10	COBRA-like protein 4	GCAGGACCACTTGGGAATGT	CCTTGGAAATGCCCATCCTT
6	*NkLAC*	c184352_g2_i3	Laccase-4-like	CCAGGAAGTGGTGGTCGTTT	ATCGGAGACGTTCGGTGCTA
7	*NkREV*	c175605_g24_i2	Homeobox-leucine zipper protein REVOLUTA-like	TGAAGTGGGTCCAGCAACAA	CTGATCGGGTGTTGCATGAG
8	*NkATHB15*	c179248_g14_i4	Homeobox-leucine zipper protein ATHB-15-like	GCTGCTCCTTGAAGCCATTG	CCAACGTCGTCTCAAGCATGT
9	*NkAS1*	c182184_g8_i3	Transcription factor AS1	CTTGAGAAAAAGGAGACGGTAACG	CTAGTTCGCTAACCAGCGTGAGT
10	*NkARF*	c181852_g1_i3	ADP-ribosylation factor	GCAGCGTCAAGACCAACCAT	GGGCTCACGTTCACGAAACT
11	*NkFAO4*	c180383_g20_i1	Long-chain-alcohol oxidase FAO4A-like	CAACCCAGCCAGATGCTCAG	GATGAGTCCGTCGTCAGGTTC
12	*NkAS2*	c181541_g7_i3	LOB domain-containing protein 6	GCCGAATATGAATGGAGAACAAT	CCTTACCGCAGTTGACCAAGA
13	*NkER*	c178690_g1_i5	LRR receptor-like serine/threonine-protein kinase ERECTA	GTTGCTCCATCATCTGAATCCA	CCGAAGGAGGCATCTACTTTTC
14	*NkAGO1*	c30628_g1_i1	Protein argonaute 1	TTTCTGCCATCTCAAACTTTTACCT	CGGAATTTTTGAATGATGTTTTCTAG
15	*NkAGO10*	c182490_g1_i14	Protein argonaute PNH1	GCTCTTATGTAATTCCTTTCTCCTCAA	TTCCTAAAGATTTTTCTCTCCTCGAA
16	*NkELF*	c185680_g1_i1	Elongation factor	GGCTTGTCCCCGAAGTTGTT	AGCACCTGGGAGACGTGATT

Note S1

To explore transcriptional changes in the different stages of *N. khasiana* leaf development, we performed k-means clustering of the significantly DEGs using Cluster 3.0. The DEGs were clustered into 12 clusters (Fig. 4A), as estimated by gap statistic using R (Fig. S7). Next, we performed functional enrichment for each cluster using BLAST2GO PRO. The enriched GO terms and representative genes for all 12 clusters are listed in Supplementary data S1. A summary of the GO terms and representative genes enriched in each stage of *N. khasiana* leaf development is given below.

Stage 1. Genes that are upregulated exclusively in stage 1, characterized by a slender structure with prominent white hairs at the apex (Fig. 2b), are grouped in cluster 8 (Fig. 4h). Cluster 8 comprises five enriched GO terms viz. 'DNA integration', 'zinc ion binding', 'nucleic acid binding', 'serine-type endopeptidase inhibitor activity' and 'aspartic-type endopeptidase activity' (Fig. 4t) and are represented by transcripts showing homology to genes such as integrase, Ty3/*gypsy* retrotransposon, GATA transcription factor, ethylene-responsive transcription factor (ERF) and several uncharacterized proteins (Table 1). In addition, DEGs of cluster 10 also showed relatively higher expression in stage 1 (Fig. 4j). Here, 16 GO terms are enriched and is represented at the top by transcripts showing homology to genes such as integrase, Ty3/*gypsy* retrotransposon, MYB transcription factors and several hypothetical proteins (Table 1). In maize, ERFs are known to show increased expression during early leaf development[1], while in Arabidopsis, nutrient-availability, as well as other signaling pathways, regulate GATA expression early in leaf development[2]. Retrotransposon promotes the retrotransposition of their genetic material into different locations within the genome and as such can generate numerous alterations in plant gene expression and function[3]. Thus, retrotransposition could play a key part in the adaptive evolution of plant traits[3]. However, transcription of these transposable elements has never been reported to cause a functional consequence. Hence, their role in pitcher formation, including *ERF* and *GATA*, may be hard to justify.

Stage 2. Genes showing relatively higher but not exclusive expression in stage 2 include cluster 1, 4 and 12 (Fig. 4a, d, l). Cluster 1 contains 56 enriched GO terms, of which photosynthesis and photosynthesis-related processes make the top list (Fig. 4m). Here, chlorophyll a/b binding proteins (*LHCB*/*CAB*), photosystem I and II reaction center subunits (*psaE, F, H, J, L, M* and *psbH, M, R*), as well as ATP synthase subunits (*atpB, E, I*), are well represented (Table 1). Cluster 4 included genes that are involved in membrane transport, dehydrogenase and hydrolase activity and several others (Table 1). Among them, the ABC transporter B gene is of interest considering its role in mediating auxin transport[4]. Cluster 12 comprises 57 enriched GO terms and is represented by

genes that are involved in chloroplast protein synthesis. These include tRNA ligases, translation initiation factor *IF1* and several plastid ribosomal proteins (Table 1). Active plastid protein synthesis is required to promote normal leaf blade development in tobacco, and the lack of it causes abnormalities due to the arrest of cell division[5]. It was also shown that lamina outgrowth in Arabidopsis is severely disrupted in the absence of plastid gene expression, caused as a result of retardation in the restriction of abaxial identity genes *FIL* and *MIR165/166*[6]. These evidences are in line with the morphological changes observed at stage 2 of *N. khasiana* leaf development, characterized by the outgrowth of the leaf base lamina (Fig. 2c).

Stage 3. The emergence of the pitcher tube becomes prominent at stage 3 (Fig. 2d). At this stage, expansion of the leaf base lamina appears completed and the lamina has become fully flattened. Significantly DEGs showing relatively higher expression in Stage 3 are grouped in cluster 2 (Fig. 4b). Twenty-four enriched GO terms are identified in cluster 2 which include 'cell wall biogenesis', 'xyloglucan metabolic process', 'xyloglucan:xyloglucosyl transferase activity', 'apoplast', 'cell wall', etc. (Fig. 4n). These GO terms are represented at the top by transcripts showing homology to genes such as xyloglucan endotransglucosylase/hydrolase (*XTH*), *COBRA-like* (*COBL*), *LACCASE* and several others (Table 1). The *XTH* gene family encodes enzymes, each of which is responsible for the metabolism of xyloglucan, a major structural component of the cell-wall matrix[7]. Xyloglucans bind cellulose microfibrils to form a cellulose-hemicellulose network necessary for the tensile strength of the cell wall. It was shown that *XTH* acts on the xyloglucans attached to cellulose microfibrils to promote cell wall loosening essential for cell growth[8]. *COBL* belonged to a small group of the *COBRA* (*COB*) gene family, known to encode glycosylphosphatidylinositol-anchored proteins[9,10]. Mutation in the *COB* gene affects the orientation of cell expansion in the Arabidopsis root[9]. It was later shown that *COB* is also required in the anisotropic expansion of cells throughout the Arabidopsis plant and act primarily to regulate microfibril orientation[11]. The oriented deposition of cellulose microfibril determines the direction of cell elongation[12]. We can assume then that the expression of *XTH* and *COBL* genes in *N. khasiana* is probably associated with proper orientation and elongation of cells at this stage of *N. khasiana* leaf development needed to control organ shape.

Stage 4. Stage 4 of the *N. khasiana* leaf development is characterized by the appearance of a flattened leaf base, a highly elongated tendril and a slightly swollen and elongated pitcher (Figs. 2e). Our k-means clustering analysis shows that the significant DEGs with increased expression exclusively at Stage 4 are grouped into 5 clusters viz. clusters 3, 5, 6, 9 and 11 (Fig. 4c, e, f, i, k). Cluster 3 comprises 23 enriched GO terms, of which 'DNA integration', 'lipid binding', 'DNA binding', 'racemase and epimerase activity', and 'acting on amino acids and derivatives' represent

the top GO terms (Fig. 4o). Genes that are enriched here include Gag-pol polyprotein, integrase, Ty3/gypsy retrotransposon, several transcription factors, DNA and RNA polymerases and Na+/H+ antiporter (Table 1). Among the enriched transcription factors, homeobox-leucine zipper genes *REVOLUTA*-like and *ATHB-15*-like are highly expressed in stage 4 and are of significant interest considering their established role in plant development (Table 1). In Arabidopsis, *REVOLUTA* (*REV*) and *ATHB15* belonged to the Class III HDZIP gene family, which also include *ATHB8*, *PHABULOSA* (*PHB*) and *PHAVOLUTA* (*PHV*), and all function in postembryonic development[13]. *ATHB15* is predominantly expressed in vascular tissue[14]; on the contrary, *REV* is expressed in various plant tissues, including vascular tissues, apical and floral meristems, and the adaxial domain of lateral organs[15,16]. Because of its restricted expression toward the adaxial portion of above-ground organs, *REV* may be involved in establishing adaxial fate in cotyledons, leaves and floral organs[15]. Another key regulator of leaf polarity enriched in cluster 3 is *AS1* (GO:0003677, Supplementary Data S1). *AS1* belongs to the ARP group of proteins that represses expression of KNOX gene during organ initiation in many diverse plant species[17,18,19,20]. The biological significance of the increased expressions of *REV*, *ATHB15* and *AS1* is discussed in the main text. Thirty-eight GO terms are enriched in cluster 5, and is represented at the top by transcripts showing homology to genes such as *DNA REPLICATION LICENSING FACTOR* (*MCM2*), *CELL DIVISION CONTROL PROTEIN 6* (*CDC6*), *KINESIN-LIKE PROTEIN 3* (*KLP3*) and several others (Table 1). The CDC6 protein along with MCM proteins confer cells the ability to initiate DNA replication[21]. In Arabidopsis, *CDC6* expression and stability are required for the induction and maintenance of endoreplication cycles during growth and development[22]. Cluster 6 comprises 40 enriched GO terms, of which 'microtubule-based movement', 'microtubule motor activity', 'kinesin complex' make the top three GO terms (Fig. 4r). Several kinesin proteins are highly enriched in cluster 6 (Table 1), some of which (e.g. *KINESIN 4*) are known to play a role in cell elongation[23]. Cluster 9 comprises 54 GO terms and is represented at the top by genes that are involved in small GTPase mediated signal transduction and GTP binding (Fig. 4u). These genes include *ADP-RIBOSYLATION FACTORS* (*ARF*s), *RAS-RELATED PROTEIN RABA*, *RAC-LIKE GTP-BINDING PROTEINS* (RACs), *SMALL G PROTEIN* (*GP*) and several others (Table 1). ARFs are core components of the vesicle transport machinery known to promote cell polarity and are also involved in the polar localization of PIN-FORMED (PIN) family auxin efflux carrier proteins[24]. They also promote cell expansion during growth and development in Arabidopsis[25]. Therefore, increased expression of these genes in *N. khasiana* may be associated with increased cell size manifested in the phenotype as a slightly longer tendril and bigger pitcher (stage 4). Cluster 11 comprises 44 GO terms and is represented at the top by genes that are involved in the regulation of transcription such

as *GATA*, *ANTHOCYANINLESS 2* (*ANL2*), transcription factor *TGA2*, homeobox-leucine zipper gene *ATHB-6-like* (*ATHB-6*), *MERISTEM L1-like* (*ML1*) and several others (Table 1). TGA2 was shown to regulate the expression of pathogenesis-related genes[26]. On the other hand, *ATHB6* is expressed in developing leaves of Arabidopsis and may have a function in cell division and/or differentiation of developing organs[27]. *ML1* is also known to promote shoot epidermal cell differentiation[28]. These evidences suggest that the increased expression of *ATHB6* and *ML1* are probably connected to the differentiation of the epidermal cells in the developing pitcher.

Stage 5. We noticed a developmental stage in which the pitcher is highly expanded and elongated with the lid remaining un-opened, and hence designated it as Stage 5 (Fig. 2a). Barring cluster 7 (Fig. 4g), the majority of significantly DEGs are downregulated at stage 5. Cluster 7 comprises 2 enriched GO terms and is represented by 2 genes which include long-chain-alcohol oxidase (FAO) and L-ascorbate oxidase (AO) (Table 1). FAO catalyzes the conversion of storage wax ester-derived fatty alcohol into fatty acid[29]. Earlier, Moreau and Huang[30] demonstrated that the wax ester acts as a food reserve for promoting seed germination and seedling growth. Because the pitcher is photosynthetically-inefficient, we can assume that FAO expressed at stage 5 of *N. khasiana* leaf development supports plant growth via mobilization of the waxy layer. Ascorbate oxidase (AO) catalyzes the oxidation of ascorbate to monodehydroascorbate (MDHA) in the apoplast. MDHA, in turn, stimulates cell growth through enhanced vacuolization[31]. Thus, it is widely believed that AO plays a role in cell elongation[32]. It is most likely that the detection of AO in *N. khasiana* at stage 5 of leaf development is associated with the expansion and elongation of the pitcher tube.

References

1. Yu, C. P. *et al*. Transcriptome dynamics of developing maize leaves and genome wide prediction of cis elements and their cognate transcription factors. *Proc. Natl Acad. Sci. USA* **112**, E2477–2486 (2015).
2. Behringer, C. & Schwechheimer, C. B-GATA transcription factors - insights into their structure, regulation, and role in plant development. *Front. Plant Sci.* **6**, 90 (2002).
3. Lisch, D. How important are transposons for plant evolution? *Nature Rev. Genet.* **14**, 49–61 (2013).
4. Zazímalová, E., Murphy, A. S., Yang, H., Hoyerová, K. & Hosek, P. Auxin transporters-why so many? *Cold Spring Harb. Perspect. Biol.* **2**, a001552 (2010).
5. Ahlert, D., Ruf, S. & Bock, R. Plastid protein synthesis is required for plant development in tobacco. *Proc. Natl Acad. Sci. USA* **100**, 15730–15735 (2003).

6. Tameshige, T. *et al*. Pattern dynamics in adaxial-abaxial specific gene expression are modulated by a plastid retrograde signal during *Arabidopsis thaliana* leaf development. *PLoS Genet.* **9**, e1003655 (2013).
7. Yokoyama, R. & Nishitani, K. A comprehensive expression analysis of all members of a gene family encoding cell-wall enzymes allowed us to predict cis-regulatory regions involved in cell-wall construction in specific organs of Arabidopsis. *Plant Cell Physiol*. **42**, 1025–1033 (2001).
8. Vissenberg, K., Fry, S. C., Pauly, M., Höfte, H. & Verbelen, J. P. XTH acts at the microfibril-matrix interface during cell elongation. *J. Exp. Bot*. **56**, 673–683 (2005).
9. Schindelman, G. *et al*. COBRA encodes a putative GPI-anchored protein, which is polarly localized and necessary for oriented cell expansion in Arabidopsis. *Genes Dev*. **15**, 1115–1127 (2001).
10. Roudier, F., Schindelman, G., DeSalle, R. & Benfey, P. N. The COBRA family of putative GPI-anchored proteins in Arabidopsis. A new fellowship in expansion. *Plant Physiol*. **130**, 538–548 (2002).
11. Roudier, F. *et al*. COBRA, an Arabidopsis extracellular glycosyl-phosphatidyl inositol-anchored protein, specifically controls highly anisotropic expansion through its involvement in cellulose microfibril orientation. *Plant Cell* **17**, 1749–1763 (2005).
12. Burk, D. H. & Ye, Z. H. Alteration of oriented deposition of cellulose microfibrils by mutation of a katanin-like microtubule-severing protein. *Plant Cell* **14**, 2145–2160 (2002).
13. Byrne, M. E. Shoot Meristem Function and Leaf Polarity: The Role of Class III HD–ZIP Genes. *PLoS Genet.* **2**, e89 (2006).
14. Ohashi-Ito, K. & Fukuda, H. HD-zip III homeobox genes that include a novel member, *ZeHB-13* (*Zinnia*)/*ATHB-15* (*Arabidopsis*), are involved in procambium and xylem cell differentiation. *Plant Cell Physiol*. **44**, 1350–1358 (2003).
15. Otsuga, D., DeGuzman, B., Prigge, M. J., Drews, G. N. & Clark, S. E. *REVOLUTA* regulates meristem initiation at lateral positions. *Plant J*. **25**, 223–236 (2001).
16. Emery, J. F. *et al*. Radial patterning of *Arabidopsis* shoots by class III HD-ZIP and KANADI genes. *Curr. Biol*. **13**, 1768–1774 (2003).
17. Waites, R., Selvadurai, H. R., Oliver, I. R. & Hudson, A. The *PHANTASTICA* gene encodes a MYB transcription factor involved in growth and dorsoventrality of lateral organs in *Antirrhinum*. *Cell* **93**, 779–789 (1998).

18. Tsiantis, M., Schneeberger, R., Golz, J. F., Freeling, M. & Langdale, J. A. The maize rough sheath2 gene and leaf development programs in monocot and dicot plants. *Science* **284**, 154–156 (1999).
19. Timmermans, M. C., Hudson, A., Becraft, P. W. & Nelson, T. ROUGH SHEATH2: a Myb protein that represses *knox* homeobox genes in maize lateral organ primordia. *Science* **284**, 151–153 (1999).
20. Byrne, M. E. *et al*. *Asymmetric leaves1* mediates leaf patterning and stem cell function in *Arabidopsis*. *Nature* **408**, 967–971 (2000).
21. Liang, C., Weinreich, M. & Stillman, B. ORC and Cdc6p interact and determine the frequency of initiation of DNA replication in the genome. *Cell* **81**, 667–676 (1995).
22. Castellano, M. M., del Pozo, J. C., Ramirez-Parra, E., Brown, S. & Gutierrez, C. Expression and stability of Arabidopsis CDC6 are associated with endoreplication. *Plant Cell* **13**, 2671–2686 (2001).
23. Kong, Z. *et al*. Kinesin-4 Functions in Vesicular Transport on Cortical Microtubules and Regulates Cell Wall Mechanics during Cell Elongation in Plants. *Mol. Plant* **8**, 1011–1023 (2015).
24. Xu, J. & Scheres, B. Cell polarity: ROPing the ends together. *Curr. Opin. Plant Biol*. **8**, 613-618 (2005).
25. Wang, Q. *et al*. A maize ADP-ribosylation factor ZmArf2 increases organ and seed size by promoting cell expansion in Arabidopsis. *Physiol. Plant.* **156**, 97–107 (2016).
26. Johnson, C., Boden, E. & Arias, J. Salicylic acid and NPR1 induce the recruitment of trans-activating TGA factors to a defense gene promoter in Arabidopsis. *Plant Cell* **15**, 1846–1858 (2003).
27. Söderman, E., Hjellström, M., Fahleson, J. & Engström, P. The HD-Zip gene ATHB6 in Arabidopsis is expressed in developing leaves, roots and carpels and up-regulated by water deficit conditions. *Plant Mol. Biol*. **40**, 1073–1083 (1999).
28. Abe, M., Katsumata, H., Komeda, Y. & Takahashi, T. Regulation of shoot epidermal cell differentiation by a pair of homeodomain proteins in Arabidopsis. *Development* **130**, 635–643 (2003).
29. Moreau, R. A. & Huang, A. H. C. Oxidation of Fatty Alcohol in the Cotyledons of Jojoba Seedlings. *Arch. Biochem. Biophys.* **194**, 422–430 (1979).
30. Moreau, R. A. & Huang, A. H. C. Gluconeogenesis from Storage Wax in the Cotyledons of Jojoba Seedlings. *Plant Physiol*. **60**, 329–333 (1977).

31. Hidalgo, A., Gonzalez-Reyes, J. A. & Navas, P. Ascorbate free radical enhances vacuolisation in onion root meristems. *Plant Cell Environ*. **12**, 455–460 (1989).
32. Pignocchi, C., Fletcher, J. M., Wilkinson, J. E., Barnes, J. D. & Foyer, C. H. The function of ascorbate oxidase in tobacco. *Plant Physiol*. **132**, 1631–1641 (2003).
